# Exploring Niches for Short-Season Grain Legumes in Semi-Arid Eastern Kenya — Coping with the Impacts of Climate Variability

**DOI:** 10.3389/fpls.2017.00699

**Published:** 2017-05-09

**Authors:** Anne Sennhenn, Donald M. G. Njarui, Brigitte L. Maass, Anthony M. Whitbread

**Affiliations:** ^1^Tropical Plant Production and Agricultural Systems Modelling, Georg-August University GöttingenGöttingen, Germany; ^2^Kenya Agricultural and Livestock Research OrganizationKatumani, Kenya; ^3^Innovations Systems in the Drylands, International Crops Research Institute for the Semi-Arid TropicsPatancheru, India

**Keywords:** APSIM, climate variability, rainfed agriculture, risk management, short-season legumes, water use efficiency

## Abstract

Climate variability is the major risk to agricultural production in semi-arid agroecosystems and the key challenge to sustain farm livelihoods for the 500 million people who inhabit these areas worldwide. Short-season grain legumes have great potential to address this challenge and help to design more resilient and productive farming systems. However, grain legumes display a great diversity and differ widely in growth, development, and resource use efficiency. Three contrasting short season grain legumes common bean (*Phaseolus vulgaris* L.), cowpea (*Vigna unguiculata* (L.) Walp.] and lablab [*Lablab purpureus* (L.) Sweet] were selected to assess their agricultural potential with respect to climate variability and change along the Machakos-Makueni transect in semi-arid Eastern Kenya. This was undertaken using measured data [a water response trial conducted during 2012/13 and 2013/14 in Machakos, Kenya] and simulated data using the Agricultural Production System sIMulator (APSIM). The APSIM crop model was calibrated and validated to simulate growth and development of short-season grain legumes in semi-arid environments. Water use efficiency (WUE) was used as indicator to quantify the production potential. The major traits of adaptation include early flowering and pod and seed set before the onset of terminal drought. Early phenology together with adapted canopy architecture allowed more optimal water use and greater partitioning of dry matter into seed (higher harvest index). While common bean followed a comparatively conservative strategy of minimizing water loss through crop transpiration, the very short development time and compact growth habit limited grain yield to rarely exceed 1,000 kg ha^−1^. An advantage of this strategy was relatively stable yields independent of in-crop rainfall or season length across the Machakos-Makueni transect. The growth habit of cowpea in contrast minimized water loss through soil evaporation with rapid ground cover and dry matter production, reaching very high grain yields at high potential sites (3,000 kg ha^−1^) but being highly susceptible to in-season drought. Lablab seemed to be best adapted to dry environments. Its canopy architecture appeared to be best in compromising between the investment in biomass as a prerequisite to accumulate grain yield by minimizing water loss through soil evaporation and crop transpiration. This lead to grain yields of up to 2,000 kg ha^−1^ at high potential sites and >1,000 kg ha^−1^ at low potential sites. The variance of observed and simulated WUE was high and no clear dependency on total rainfall alone was observed for all three short-season grain legumes, highlighting that pattern of water use is also important in determining final WUE_biomass_ and WUE_grain_. Mean WUE_grain_ was lowest for cowpea (1.5–3.5 kg_grain_ ha^−1^ mm^−1^) and highest for lablab (5–7 kg_grain_ ha^−1^ mm^−1^) reflecting the high susceptibility to drought of cowpea and the good adaptation to dry environments of lablab. Results highlight that, based on specific morphological, phonological, and physiological characteristics, the three short-season grain legumes follow different strategies to cope with climate variability. The climate-smart site-specific utilization of the three legumes offers promising options to design more resilient and productive farming systems in semi-arid Eastern Kenya.

## Introduction

Semi-arid areas of sub-Saharan Africa are among the most vulnerable regions worldwide to the impacts of climate variability and change (Slingo et al., 2005; Boko et al., 2007; Challinor et al., 2007; Thornton et al., 2011). Statistics on temperature and precipitation patterns reveal, for instance, that most of Eastern Africa became warmer in the last century and that rainfall exhibits an increased inter- and intra-seasonal variability (Boko et al., 2007; Challinor et al., 2007; Cooper et al., 2009). Furthermore, erratic weather patterns characterized by cycles of droughts have become more frequent. In semi-arid Eastern Kenya annual rainfall ranges from 330 to 1,260 mm with 85% falling during the growing seasons: the short rains from October to February and the long rains between March and May; and with a coefficient of variation exceeding 40% (Rao and Okwach, 2005). Whilst efforts have been made to improve seasonal climate forecasts and apply this information to reduce risk in rainfed agricultural production systems in semi-arid Eastern Kenya (Hansen et al., 2009) there remain many barriers that hinder small-scale farmers from benefiting from this technology. First, the lack of spatial coherence of rainfall intensities in semi-arid Eastern Kenya contribute to high sampling error and comparatively low reliability even when downscaled (Rao and Okwach, 2005). Second, the tremendous spatial variability in soil properties in this region adds to the complexity of designing more resilient cropping strategies and influences the willingness of farmers' to respond to season forecast. Third, the inadequate communication of the forecast information to the rural areas hinders their application and possible positive impact. Therefore, tremendous investment in innovative agricultural extension services is required to overcome these constraints.

While these tactical approaches to minimize climate risk have so far had limited impacts and acceptance, redesigning farming systems to better cope with climate risk is a strategic approach that must also be considered. In particular, legumes display a wide agro-morphological diversity with great potential for adaptation to semi-arid environments. The benefits of green manure, grain and fodder legumes for the farmer, farming systems, environment, and economy have been reported in manifold publications and are widely acknowledged in smallholder farming systems, including those of Eastern Kenya (Graham and Vance, 2003; Siddique et al., 2012; Foyer et al., 2016). In particular, locally well-adapted short-season grain legumes from semi-arid areas, such as common bean [*Phaseolus vulgaris* (L.)], cowpea [*Vigna unguiculata* (L.) Walp.], and lablab [*Lablab purpureus* (L.) Sweet] offer new possibilities for sustainable farming with increased uncertainties in risk-prone environments, including new management options addressing the challenges of changing growing season characteristics. However, there is limited information available on growth and development and resource use and use efficiency. The comparative water use and use efficiencies (WUE) of short-season grain legumes in semi-arid environments is particularly crucial for the production success in rainfed production systems (Asseng et al., 2001). Furthermore, a general problem remains the lack of knowledge on the use of climate information and the adaptation of agricultural interventions, such as short-season grain legume varieties to improve agricultural production. The possible impact of legumes to contribute to increased sustainability in risky environments has not yet been studied well and most research from semi-arid Eastern Africa focuses on the quantification of climate uncertainties and their impact on maize production only.

Simulation models have proven to be excellent tools to explore the potential of certain crops and cropping strategies in diverse smallholder farming systems and different environments (Whitbread et al., 2010). As farming systems in semi-arid areas are highly heterogeneous, simulation models may help to address the complexity of these systems, which is difficult through classical agronomic experiments alone (Robertson et al., 2001; Holzworth and Huth, 2009; Whitbread et al., 2010). One of the most applicable models to better understand plant growth and development in response to the environment has been the Agricultural Production Systems sIMulator (APSIM) framework (Keating et al., 2003; Holzworth et al., 2014). APSIM simulates biophysical—key soil and crop—processes for a wide range of crops and environmental conditions. The model was, however, well-calibrated for resource-constrained and risky environments of semi-arid smallholder farming systems (Whitbread et al., 2010). As a tool, modeling frameworks may be used to address primary challenges and limitations such as inter- and intra-seasonal rainfall variability as well as the variation in crop response to diverse soil types and agronomic management (Whitbread et al., 2010). Furthermore, the APSIM maize model, for instance, was widely validated against a wide range of datasets from Eastern Kenya (Keating et al., 1992) and is, therefore, well-suited for simulation studies in semi-arid areas. With the use of simulation models, such as APSIM, biomass and grain production as well as the water use of promising crops can be extrapolated. A major limitation to this effort has been the limited range of legumes that have been calibrated and validated within the crop modeling framework, in particular short season types. The application of well-calibrated crop growth models, however, could help to estimate their production potential across different sites and soil conditions, as well as the impact of different management interventions. Furthermore, this would allow us to better assess the interaction of phenology with patterns of water use and WUE. This is of great interest in order to develop crop adaptation strategies in terms of combating climate variability.

With this background we hypothesize, first, that climate variability and uncertainties have increased over the past decades and consequently associated risk for crop production systems in semi-arid Eastern Kenya. To test this hypothesis long term weather data from semi-arid Eastern Kenya was revised and analyzed. Secondly, we hypothesize, that short-season grain legumes have great potential to address these challenges and help design more resilient and productive farming systems. To test this hypothesis, first, water use and water-use efficiency of the selected short-season grain legumes were quantified from comprehensive datasets derived from field experiments in Eastern Kenya. Secondly, these datasets were used to calibrate and validate APSIM to simulate growth and development of short-season-grain legumes under semi-arid conditions. Third, with the validated models, water use and water use efficiency (WUE) as well as the productivity of short-season grain legumes were simulated for different sites and soil types along the environmental gradient of Machakos-Makueni in semi-arid Eastern Kenya.

## Materials and methods

### Description of the study area

The study area was located in the predominantly semi-arid Eastern Province of Kenya and covers the Machakos—Makueni transect (Appendix 1), which forms an environmental gradient of decreasing altitude, increasing temperatures, and decreasing moisture from the northwest to the southeast; resulting in a wide range of agro-ecological conditions (Jaetzold et al., 2006). The physical settings (topography and elevation) mainly influence the quantity and distribution of rainfall. The precipitation pattern is bimodal, with the locally known “short rain” season (SR) from October to February and a so-called “long rain” season (LR) between March and June. The amount of rainfall decreases along the transect from Machakos to Makueni: total annual averages are between 1,300 and 350 mm (Gichuki, 2000). Mean annual temperatures range from 17 to 24°C. Farm size and population density across the research area are mainly driven by the availability of water and soil quality to sustain agriculture. In medium-potential areas of the upper midlands in the northwest, farm size is rather small ranging from 0.5 to 1.5 ha, whereas in the low-potential areas of the lower midlands in the southern parts, farm size is comparatively large: 3–5.5 ha (Jaetzold et al., 2006). Land use and livelihood are dominated by small-scale mixed farming systems: based on rainfed crop production combined with different levels of livestock rearing. Main crops grown on the mainly family-owned farm land are maize and common bean (Muhammad et al., 2010).

### Analysis of climate variability

Daily weather data was obtained from the meteorological stations of the centers and sub-centers of the Kenya Agricultural and Livestock Research Organization [KALRO, formerly Kenya Agricultural Research Institute (KARI)] in the study area including Katumani, Kampi ya Mawe, and Makindu. Radiation data was partly obtained from the National Aeronautics and Space Administration (NASA) database for Climatology Resource for Agroclimatology (http://power.larc.nasa.gov/cgi-bin/cgiwrap/solar/agro.cgi?email=agroclim@larc.nasa.gov).

The temporal rainfall variability for the three selected sites within the study area of Eastern Kenya (Table 1) was determined by calculating the coefficient of variation (CV) as the ratio of standard deviation to the mean annual rainfall in a given period. Further values of accumulated rainfall (monthly, seasonal, annual), number of rain days, rainfall intensity (amount of rainfall per day), start of growing season, end of growing season, length of growing season, and dry spell probability were determined. To estimate the behavior of temperature over time a linear model was adjusted to the annual means using generalized least squares (function gls, R package nlme) (Pinheiro et al., 2014). The correlation structure of the underlying data was considered through the incorporation of the potential autoaggressive structure in residuals defined by an autoregressive process of order 1 (function corAR1, R add-on package nlme) Pinheiro et al., 2014). Additionally, number of days with t_max_ > 25°C were determined (Klein Tank et al., 2009). The growing season characteristics (growing season start, and length) were calculated according to Stern et al. (1982). The 1st of October was set as the earliest possible planting date for the short rain season and the 1st of March for the long rain season (Muhammad et al., 2010; Stern and Cooper, 2011). The dry spell probability at each site was estimated on the basis of generalized linear models for binomial responses using the complementary log-log link function selected according to Akaike (1973) information criterion of dry spells >5, 7, 10, or 15 days. The smooth effect function for Julian day of year were specified according to cyclic P-splines (Eilers and Marx, 1996). All calculations were performed using R 3.1.1 (R Core Team, 2014) and, in particular, package mgcv (Wood, 2011). Two stations (Katumani and Kampi ya Mawe) were selected, which have relatively long periods (at least 30 years) of data with no more than 5% missing values for rainfall and temperature to obtain detailed climate variability analyses, including growing season characteristics, dry spell probability and temperature trends (Table 1).

**Table 1:** **Geographical information as well as, availability of rainfall, temperature, and radiation data for the study sites in Eastern Kenya**.

**Site**	**Latitude**	**Longitude**	**Elevation (m asl.)**	**Data gathering period (years)**		
				**Rainfall**	**Temperature**	**Radiation**
Katumani	1°34′56″S	37°14′43″E	1,592	1961–2013	1981–2013	1981–2013
Kampi ya Mawe	1°51′0″S	37°40′0″E	1,150	1961–2012	1970–2012	1981–2012
Makindu	2°16′58″S	37°49′58″E	1,070	1997–2013	1977–2013	1997–2013

### Agronomic trials

Agronomic data of common bean (navy bean), cowpea and lablab were derived from two major experiments (plant density and water response trial) conducted on the KARI Katumani research station during the short rain season 2012/13 and 2013/14 described in detail by Sennhenn (2016). Locally adapted and commonly used short-season varieties recommended by KARI for cultivation in small-scale farming systems in semi-arid areas were used in the experiments; KAT X56 for common bean, M66 for cowpea and DL1002 for lablab (KARI, 2006) (Table 2). Sowing was carried out at the onset of the rains on the 14th of November in 2012 and on the 5th of November in 2013.

**Table 2:** **Description of phenological development and growth characteristics of short-season grain legumes in semi-arid Eastern Kenya**.

**Species**	**Variety**	**Time to 50% flowering [DAP]^a^**	**Time to physiological maturity [DAP]^a^**	**Growth habit**	**Canopy architecture**
Bean	KAT X56	37–41	69–78	Bushy	Compact, small
Cowpea	M66	61–66	84–92	Spreading	Widespread, large
Lablab	DL1002	57–60	98–104	Bushy	Compact, large

a *DAP–days after planting*.

Experiment 1, the plant density trial was designed to provide data on legume phenology as well as biomass and grain yield development in response to plant density. Therefore, the legumes were sown at three different plant densities; “medium” (bean and cowpea: 10 plants m^−2^, lablab: 4.17 plants m^−2^) following the recommendations by KARI for farming in semi-arid areas (KARI, 2006), while “high” was double, and “low” only half of the recommended density.

Experiment 2, the water response trial aimed to deliver data on biomass development and water use as well as soil moisture dynamics in respect to water availability. All three short-season grain legumes were grown under optimal (“medium”) plant density with three water treatments; purely rainfed, partly irrigated (total 50 mm of water per week with additional drip irrigation till bud formation, i.e., onset of flowers), fully irrigated (total of 50 mm of water per week with additional drip irrigation throughout the growing period) (Table 1). A summary table of all treatments included in the two experiments, as well as an overview of the corresponding plant densities and water treatments can be found in the Supplementary Material (Appendix 3). Throughout the experiments, the phenological development was monitored (in days after planting, DAP), and biomass and grain yield (in kg/ha) development were measured in 2-weekly intervals.

Leaf area index (LAI) were measured in intervals of 7–10 days (dependent on daily cloudiness) using an AccuPAR LAI ceptometer (Decagon Devices, model LP-80) (Sennhenn, 2016).

The trials were located on fairly well-drained reddish brown chromic Luvisols with a clay texture throughout the profile and an increased sand content in the surface layer (Jaetzold et al., 2006). The soil was slightly acid to neutral (pH 5.5–7), and was low in plant available nitrogen, phosphorus, calcium, and zinc and had low organic carbon content (OC ≤ 1%). At sowing, 50% flowering, physiological maturity and throughout the experiments at 2-week intervals, soil moisture in each subplot was monitored (gravimetrically depth-wise for the top four layers: 0–15, 15–30, 30–60, 60–90 cm).

From the water response trial, water use and use efficiency were examined: on the basis of the measured data, evapotranspiration (*E*_*t*_) was determined based on the hydrological approach using the soil water method (Muchow, 1985; Rana and Katerji, 2000):

(1) $$\begin{array}{r} E_{t}=\Delta W+P+I-D-R \end{array}$$

Where ΔW is the change in water stored over the period considered, *PP* is the precipitation and *II* is the amount of irrigation applied. Drainage (*D*) and Runoff (*R*) were estimated with the help of a simulation model; side- and season specific drainage and runoff were assessed using soil module SoilWat, APSIM (Keating et al., 2003). The above-ground biomass and grain yield dry matter at harvest were divided by *E*_*t*_ to provide values on respective water use efficiencies, *WUE*_*DM*_ and *WUE*_*Yield*_:

(2) $$\begin{array}{rcl} WUE_{biomass} & = & \frac{Biomass}{E_{t}} \end{array}$$

(3) $$\begin{array}{rcl} WUE_{grain} & = & \frac{Grainyield}{E_{t}} \end{array}$$

### APSIM model calibration and validation

The legume crop modules of common bean (navy bean), cowpea and lablab were calibrated and validated within the APSIM (APSIM 7.5) framework for short-season varieties grown under semi-arid conditions. Generally, input data required for the model are crop management information, cultivar specific parameters (genetic coefficient), soil properties and daily weather records.

#### Crop

Crop management and cultivar information for the three short-season grain legumes were derived from the two field experiments (plant density and water response trials) described above. For calibration purposes, the treatments with optimal planting density (bean and cowpea: 10 plants m^−2^ lablab: 4.17 plants m^−2^) grown under fully irrigated conditions (up to 50 mm per week with additional irrigation) from the experiment 2, water response trial, from 2012/13 were used. Cultivar-specific parameters, important for the calibration procedure, which were determined to specify and quantify growth and development of the short-season grain legumes, included harvest index (HI), daily increase in HI and thermal times to reach certain development stages. The remaining data from the experiment 1 (plant density trial) experiment 2 (water response trial) from 2012/13 and 2013/14 were used for model validation. In terms of validation, this study focuses on phenology, soil moisture as well as on biomass and grain yield. A detailed description of the crop calibration and validation procedure can be found in Sennhenn (2016).

#### Soil

Soil parameters used for the model were derived from on-site soil characterization (Sennhenn, 2016) and published soil characterization for sites at the KARI Katumani research station in Machakos, Kenya (Gicheru and Ita, 1987). The two parameters that determine first *U*(*U*) and second stage (*c*) of soil evaporation were set to 4 and 2 mm day^−1^, respectively, representative for a sandy loam soil. Further the SOILWAT and SOILN modules were parameterized as described in Table 3 with further detail found in Sennhenn (2016). Plant available water (PAW) was estimated from the soil moisture and the species-specific crop lower limit. Rooting depth was 105, 120, 105 cm for common bean, cowpea and lablab respectively.

**Table 3:** **Layer soil type parameters used by APSIM-SOILWAT module: bulk density (BD), soil water content at air-dry (AIR_DRY), 1.5 MPa tension (LL15), drained upper limit (DUL), saturation (SAT), and species-specific crop lower limit (CLL) at the experimental site KARI Katumani, Kenya**.

**Depth (cm)**	**0–15**	**15–30**	**30–60**	**60–90**	**90–120**	**120–150**	**150–180**
BD (g cm^−3^)	1.57	1.57	1.54	1.5	1.51	1.51	1.51
AIR_DRY^a^ (cm cm^−1^)	0.020	0.052	0.085	0.099	0.099	0.099	0.099
LL15^a^ (cm cm^−1^)	0.039	0.072	0.085	0.099	0.099	0.099	0.099
DUL (cm cm^−1^)	0.190	0.210	0.300	0.350	0.350	0.350	0.350
SAT (cm cm^−1^)	0.378	0.378	0.389	0.404	0.4	0.4	0.4
CLL (cm cm^−1^)							
Common bean	0.039	0.072	0.122	0.138	0.138	0.138	0.138
Cowpea	0.039	0.072	0.085	0.099	0.099	0.099	0.099
Lablab	0.039	0.072	0.100	0.110	0.120	0.120	0.120

a *adapted from Gicheru and Ita (1987) similar to APSIM soil file: “Chromic Luvisol, Katumani Research Station” from the international. APSIM soil file database for Kenya*.

#### Weather

**Weather**, at the study site, minimum and maximum temperature, rainfall (+irrigation) were recorded on a daily basis. Further, solar radiation records were obtained from the meteorological station at KARI Katumani (for details see Supplementary Material, Appendix 2).

### Simulation of the agricultural potential and water use efficiency

After calibration and validation of the models, a multi-year simulation with historical weather data (Table 1) was performed to analyze the agricultural potential, including grain yield as well as water use and use efficiency of the three short-season grain legumes. The simulations were carried out for three sites, similar to the sites used for the climate variability analysis described in the previous section on the basis of the same historical weather data (Table 1). Three soils representing three major soil groups in Eastern Kenya available in the APSIM soil toolbox were chosen to examine the effect of available water-holding capacity of the soil in interaction with site-specific rainfall characteristics and crop management (Table 4). The soils mainly differ in texture and plant available water content (PAWC). Soil water was reset to the lower limit (LL) on 1st of October. Between the short rain season (October–February) and the long rain season (March–June), soil water was not reset since the long rain season partly depends on residual soil moisture of the previous short rains. The initial nitrogen content was similar for all soils and reset at the beginning of each cropping period (1st of October and 1st of March) to eliminate a bias for nutrient availability on crop growth and development (mineral *N* in the profile 0–180 cm was 48.5 kg ha^−1^).

**Table 4:** **Detailed description of soils used for the simulation study and their characteristics, including soil texture, plant available water capacity (PAWC) in mm, pH, and organic carbon content in %**.

**Soil ID**	**APSIM soil name**	**Soil texture**	**USDA soil classification**	**PAWC^a^ (mm)**	**pH^b^**	**Organic carbon^b^ (%)**
High PAWC	Chromic Luvisol, Katumani, Kenya	Sandy clay	Luvisol	164	6.0	0.92
Medium PAWC	Clay loam, Alfisol, Masii district, Kenya	Clay loam	Alfisol	137	6.0	1.10
Low PAWC	Sand, Alfisol, Masii district, Kenya	Sand	Alfisol	87	6.0	0.60

a *Plant available water capacity*.

b *Measured for the 0–150 mm soil depth*.

Sowing time was controlled by a sowing rule aligned with the start of the season. Sowing was realized after 1st of October during the short rain season and after 1st of March for the long rain season and did not occur until 20 mm of rainfall accumulates over 3 consecutive days. Growth and development of short-season varieties of common bean, cowpea and lablab (Table 2) were simulated for both the growing period of the short rain season and the long rain season. Plant density was set similar to the experiment described above. All planting rules represent current “best farmer's practice.” Phenological development (time to 50% flowering and physiological maturity), biomass and grain yield production were simulated. Water-use efficiency was estimated according to site- and soil-specific evapotranspiration relative to crop productivity (Monteith, 1988). Therefore, potential evapotranspiration in the APSIM model was determined as described by Holzworth et al. (2014) and Probert et al. (1998). WUE_biomass_ and WUE_grain_ were defined as the ratio of total biomass and grain yield, respectively, to evapotranspiration (E_t_) between sowing and harvest (Equations 2, 3), calculated from the model output.

### Statistical analysis

To analyze the trial data, biomass and grain yield as well as water use indices were compared among legume species and treatments, using analysis of variance (ANOVA). Each field trial and season were analyzed separately because of environmental variations. Within the species, treatment effects were characterized using test of significance *post-hoc* multiple comparison Turkey test. To assess intra-specific differences in water-use efficiency, data corresponding to the rainfed treatment only were extracted and least significant differences (LSD) were computed using R 3.1.1 (R Core Team, 2014).

The model validation was performed with the dataset derived from the plant density and water response trial (Supplementary Material, Appendix 3) for flowering and maturity dates, soil moisture content of the soil profile as well as biomass and grain yield. Measured and predicted data were compared graphically and analyzed statistically. The root mean square error (RMSE) and the modeling efficiency (EF) were computed (Willmott, 1981), (Equations 4, 5) as follows:

(4) $$\begin{array}{rcl} RMSE & = & \sqrt{\sum_{i\;=\;1}^{i\;=\;n}\frac{{\left(P_{i}-O_{i}\right)}^{2}}{n}} \end{array}$$

(5) $$\begin{array}{rcl} EF & = & 1-\left[\frac{\sum_{i\;=\;1}^{i\;=\;n}{\left(P_{i}-O_{i}\right)}^{2}}{\sum_{i\;=\;1}^{i\;=\;n}{\left(O_{i}-\bar{O}\right)}^{2}}\right] \end{array}$$

Root mean square error (RMSE) with *P*_*i*_, predicted value, *O*_*i*_, observed value, $\bar{O}$, mean of the observed values and *n*, number of observation. RMSE and EF were calculated for biomass and grain yield. Additionally, for comparison, the traditional *R*^2^ regression statistic (least-squares coefficient of determination) was determined.

## Results

### Climate variability

Within the Machakos—Makueni transect in Eastern Kenya the spatial distribution of rainfall is linked to physical settings, mainly topography and elevation, with the highest mean annual rainfall records for Katumani (996 mm), medium for Kampi ya Mawe (640 mm), and the lowest for Makindu (545 mm) (Table 5). The rainfall pattern is bimodal across the study area and the so-called short rains (October–February), received on average more rain than the growing period of the long rains (March–June). The seasonal variation in rainfall was very high for all sites (Table 5).

**Table 5:** **Rainfall and the respective coefficient of variation (CV) for three study sites in Eastern Kenya, including Katumani, Kampi ya Mawe (KyM), and Makindu calculated from different datasets as indicated in Table 1**.

**Site**	**Temperature (°C)**			**Rainfall (mm)**					
	**Mean**	**Maximum**	**Minimum**	**Annual**		**Short rain**		**Long rain**	
				**Mean**	**CV (%)**	**Mean**	**CV (%)**	**Mean**	**CV (%)**
Katumani	21.0	26.2	15.8	695.8	28.0	391.5	42.4	290.6	41.1
KyM	23.0	28.9	17.1	639.6	35.9	383.7	41.0	247.7	51.9
Makindu	22.2	27.7	16.7	544.5	30.1	281.3	39.9	227.4	34.6

**Figure 1:**
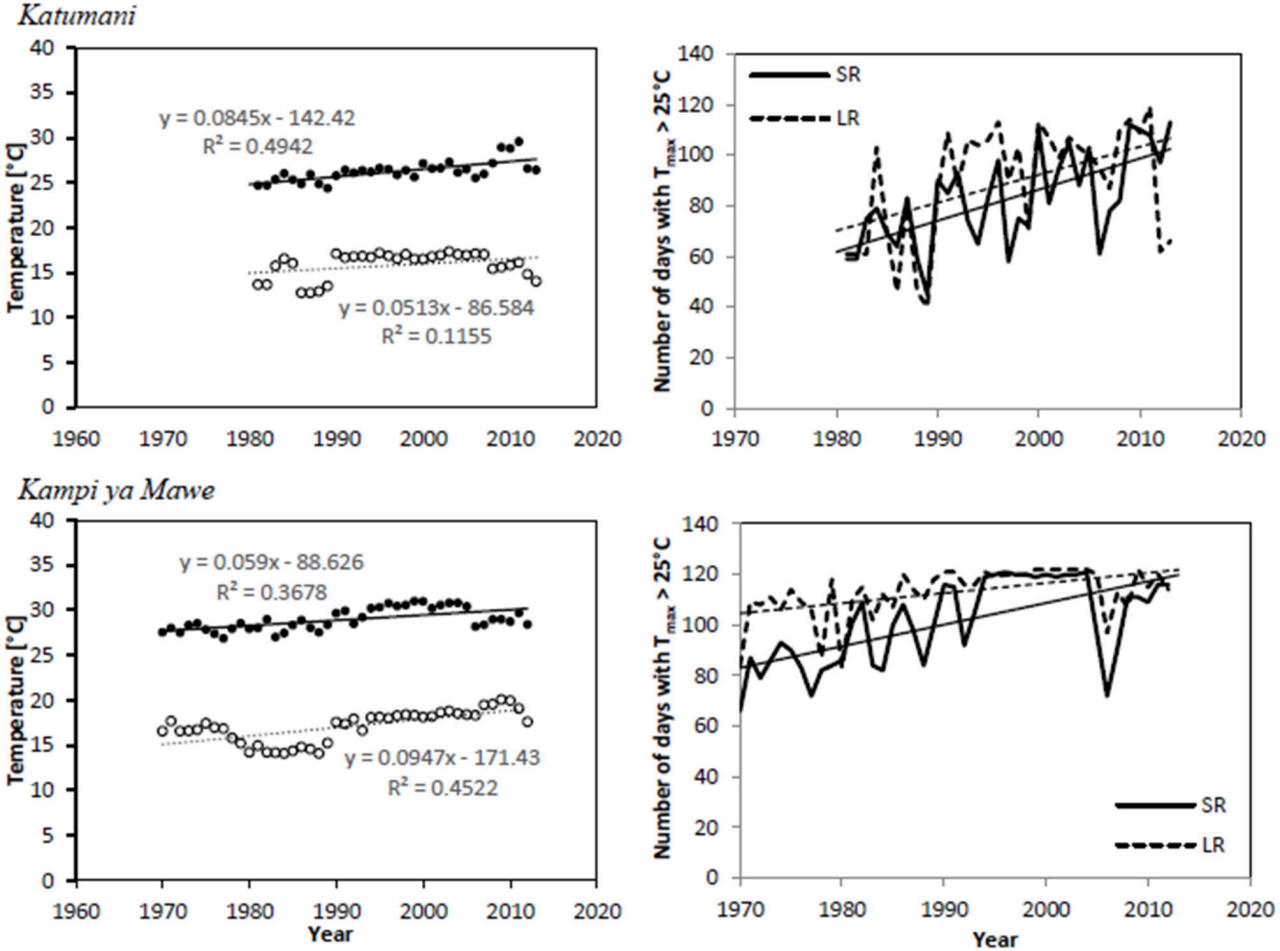
**Time series and trends for minimum (◦) and maximum (•) temperatures as well as number of days with maximum temperatures > 25°C for the growing period of the short rain (SR,—) and long rain (LR,−−−) at Katumani and Kampi ya Mawe, Eastern Kenya**.

For both sites Katumani and Kampi ya Mawe, mean annual minimum and maximum temperatures showed a significant warming trend over the years (Table 5 and Figure 1). The comparatively pronounced trend of increasing mean maximum temperatures in Katumani is partly driven by a large increase in days with maximum temperatures above 25°C, which was observed for both growing periods (the short rain and the long rain) during the last three decades (Figure 1).

**Figure 2:**
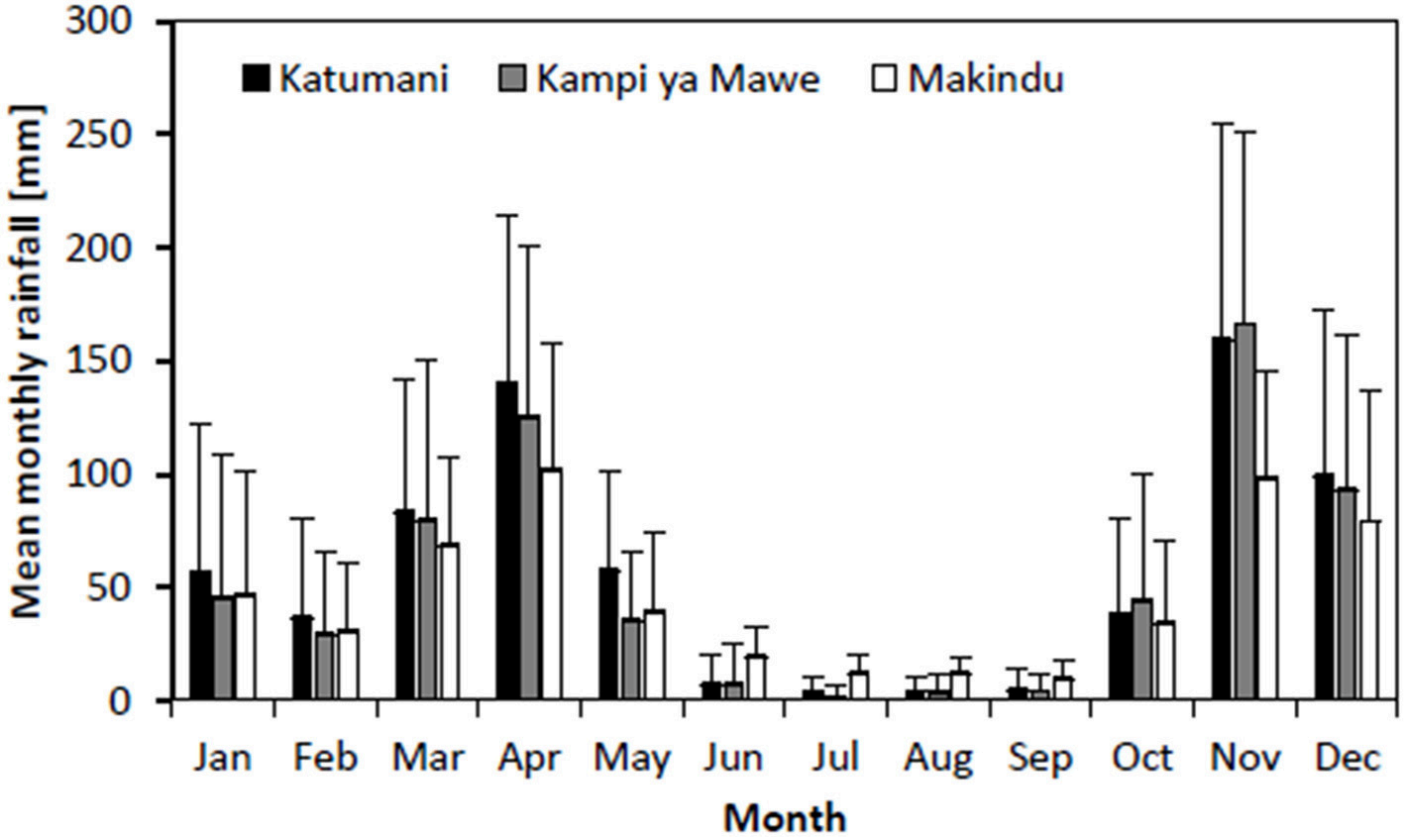
**Monthly rainfall distribution with standard deviation for Katumani (period: 1961–2013), Kampi Ya Mawe (period: 1961–2012), and Makindu (period: 1997–2014) Eastern Kenya**.

Analyses for the seasonal rainfall of the two selected stations, Katumani and Kampi ya Mawe, indicated that rainfall during the growing seasons in Eastern Kenya generally exhibited a high inter-seasonal variability (Figure 2, Table 5). Furthermore, results showed that both rainfall intensity (Katumani SR: 8.3 LR: 7.9 mm per rainy day; Kampi ya Mawe SR: 8.8, LR: 8.1 mm per rainy day) and mean number of rainy days per growing period (Katumani SR: 43, LR: 32 rainy days per season; Kampi ya Mawe SR: 37 LR: 25 rainy days per season) were higher during the short rain growing period. Furthermore, results of the historical weather data highlighted that not only the total seasonal rainfall decreased over the last decades, in particular in Kampi ya Mawe, but also the rainfall intensity per rainy day (data not shown).

**Figure 3:**
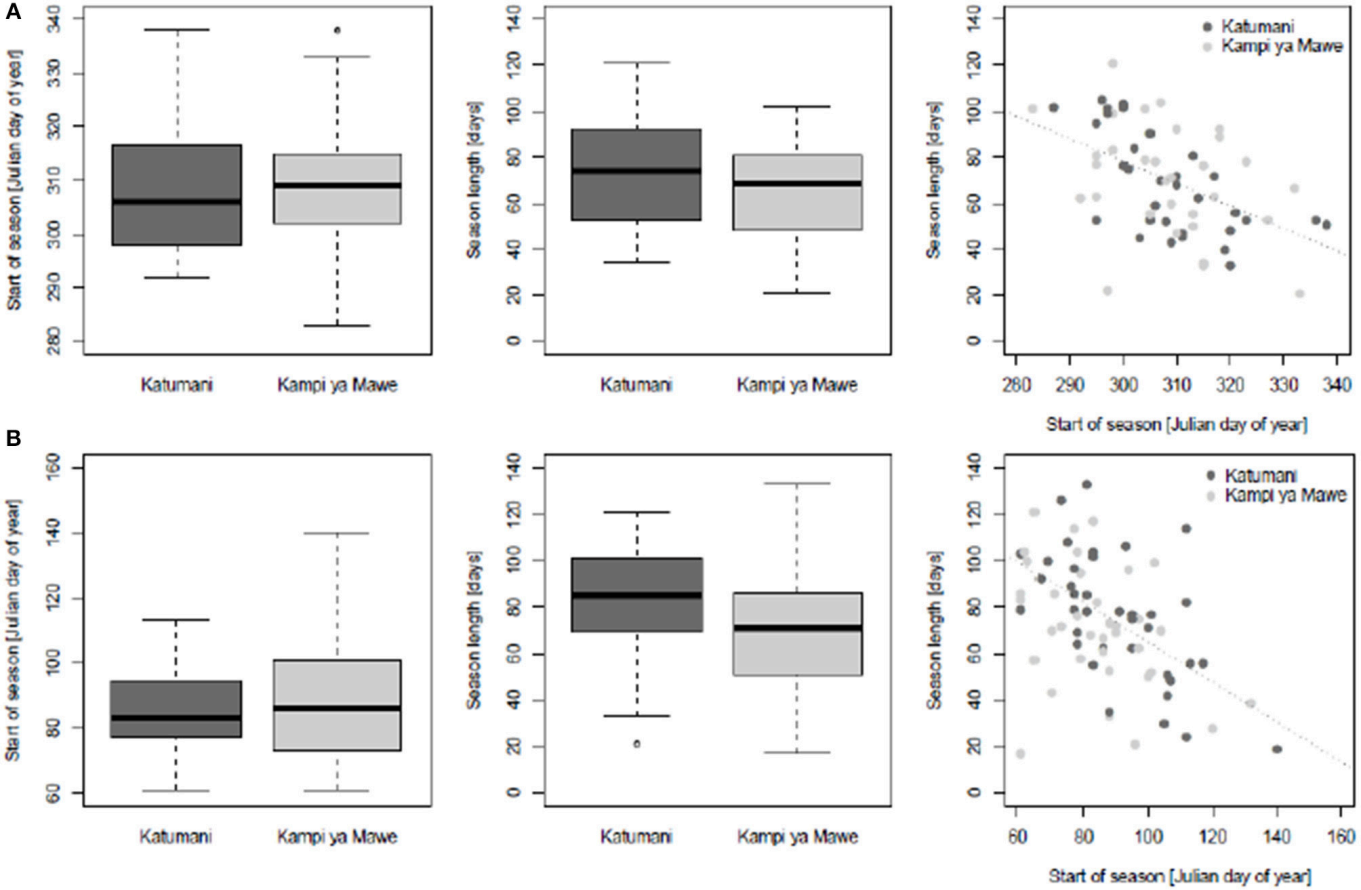
**Boxplots representing characteristics of growing season length in Katumani and Kampi ya Mawe, Eastern Kenya, including start of growing season (day of the year, DOY) and growing season length (days) as well as the relationship between start of growing season and growing season length for the short rain (A)** and the long rain **(B)** season according to data as described in Table 1.

The analysis of the start of season and the length of the season showed a high inter-annual variability for both sites Katumani and Kampi ya Mawe (Figure 3). The 25 and 75% percent quartile—a measure of the long-term variability—was particularly wide for the short rain season start in Katumani. Results further show, that the later the seasons starts the shorter is their expected length. Similar trends were observed for the long rain season. The observed variability in the start of season (inter quartile range: Julian day number 73–101) and length (inter quartile range: 51–86 days) was, however, much higher for Kampi ya Mawe compared to Katumani for the long rain season. The high degree of variability in the start of each growing season and growing season length highlight the high degree of uncertainty associated with cropping activity planning and adds to the risks for farming practice in Katumani and Kampi ya Mawe. The dry spell analysis clearly showed that the probability of occurrence of longer dry spells was particularly distinct from July until September and at the end of February for the short and long rainy season, respectively. Even within the rain seasons, the probability of dry spells longer than 5 and 7 days was higher in Kampi ya Mawe (SR: 18, LR: 12%) than in Katumani (SR: 15, LR: 9%) and particularly high for the rather unreliable long rain.

### Agronomic trials

#### Water use and use efficiency

Common bean proved to be a true short-season crop, and first flowering was observed already about 36 DAP with grains ready to harvest after 75 DAP. Lablab flowered earlier (43–47 DAP) than cowpea (47–54 DAP), however, time to physiological maturity was longest for lablab with 98–104 DAP. Water use was always lowest for common bean, independent of the water treatment applied due to the fast phenological development, and highest for lablab due to its long maturity time (**Table 7**). Biomass production and grain yields were dissimilar in the two distinct seasons, mainly caused by differences in total seasonal rainfall and temporal rainfall distribution over the growing seasons. In the growing period of 2012/13, rain was below the long-term average (Rao and Okwach, 2005; Claessens et al., 2012) with 262 mm only, nevertheless relatively evenly distributed between November and January but no rain in February. During the short rain of 2013/14, total rainfall was about long-term average (354 mm), but very unevenly distributed (220 mm falling between end-November to end-December, and a long in-growing period dry spell from 22nd December to 6th February) (Supplementary Material, Appendix 2).

Under rainfed conditions the accumulated biomass and grain yield were always lowest for common bean (Table 6). However, biomass and grain yield of common bean was fairly similar for the different treatments, under rainfed conditions and fully irrigated, in both seasons, indicating relatively stable yields independent of in-season rainfall amount and distribution. Biomass and grain yields for cowpea (grain yield: ~1,500 kg ha^−1^) and lablab (grain yield: 1,880 kg ha^−1^) were higher in the wetter season of 2013/14. The growing period of the short rains 2013/14 was characterized by intensive rainfall from end-November to end-December and a very long dry spell in January. In this season the yield increase with applied irrigation was significant and highest for common bean (+100%) and less, however, still significant for cowpea (+47%) and lablab (+26%). This is an indication for a higher drought compensation capability by cowpea and lablab in comparison to the truly short-season legume common bean. WUE in terms of biomass production and grain yield was always highest without supplementary irrigation, except for cowpea in 2012/13 (Table 6). Similar to the trends in biomass and yield development for the short-season common bean, WUE_biomass_ was higher in 2012/13 in comparison to the 2012/14 season, whereas the opposite was true for cowpea and lablab. Similar was observed for WUE_grain_. During the comparatively dry growing period of the 2012/13 short rain, WUE_grain_ was highest for common bean (5.9 kg ha^−1^ mm^−1^) without additional irrigation but not significantly different from cowpea (5.0 kg ha^−1^ mm^−1^) and lablab (5.1 kg ha^−1^ mm^−1^). However, in 2013/14, WUE_grain_ was significantly increased for cowpea (5.9 kg ha^−1^ mm^−1^) and lablab (6.5 kg ha^−1^ mm^−1^) in comparison to common bean (4.0 kg ha^−1^ mm^−1^) under rainfed conditions.

**Table 6:** **Irrigation, rainfall, water use (E_***t***_), and water use efficiency for biomass production and grain yield of short-season legume species grown under different water regimes in Machakos, Eastern Kenya during the short rains of 2012/13 and 2013/14**.

**Season**	**Species**	**Water regime**	**Irrigation (mm)**	**In-crop rainfall (mm)**	**Irrigation + rainfall (mm)**	**E_t_(mm)**	**Total biomass at harvest (kg DM ha^−1^)**	**Grain yield (kg ha^−1^)**	**WUE_biomass_ (kg ha^−1^ mm^−1^)**	**WUE_grain_[kg ha^−1^ mm^−1^]**
2012/13	Bean	Fully irrigated	270	156	426	481	3,638	1,888	7.6	3.9
		Rainfed	0	156	156	187	2,182	1,107	11.7	5.9
	Cowpea	Fully irrigated	300	190	490	578	5,629	3,061	9.7	5.3
		Rainfed	0	190	190	277	2,574	1,385	9.3	5.0
	Lablab	Fully irrigated	345	190	535	609	3,652	1,933	6.0	3.2
		Rainfed	0	190	190	243	2,966	1,234	12.2	5.1
*L.S.D. [water treatment: rainfed] *P* = 0.5*							*408*	*245*	*1.9*	*1.1*
2013/14	Bean	Fully irrigated	240	259	499	503	3,335	1,956	6.6	3.9
		Rainfed	0	259	259	245	1,762	978	7.2	4.0
	Cowpea	Fully irrigated	330	259	589	596	4,487	2,210	7.5	3.7
		Rainfed	0	259	259	256	3,030	1,512	11.8	5.9
	Lablab	Fully irrigated	345	339	684	635	5,474	2,352	8.6	3.7
		Rainfed	0	339	339	290	3,906	1,873	13.5	6.5
*L.S.D. [water treatment: rainfed] *P* = 0.5*							*787*	*367*	*3.0*	*1.4*

*L.S.D.–Least Significance Difference*.

### APSIM model calibration and validation

Cultivar-specific parameters presented in Table 7, which have been adjusted within the APISM calibration procedure, include HI and daily potential increase in HI as well as thermal time from emergence to various developmental stages. Results show that the cultivar-specific parameters (Table 7) were selected well to account for the high-yielding and short-season characteristics of the short-season grain legumes tested (Appendices 4, 5). The phenological development was captured excellent with a very high accuracy. Furthermore, the total biomass development was pictured very well by the calibrated model, indicating a god coverage of the morphological and physiological characteristics of the different legume species. Details for the model calibration are shown in the Supplementary material (Appendices 4, 5).

**Table 7:** **Cultivar-specific parameters for different short-season grain legume species common bean, cowpea, and lablab use to calibrate the Agricultural Production Systems sIMulator (APSIM)**.

**APSIM parameter description**	**Units**	**Legume species**		
		**Bean**	**Cowpea**	**Lablab**
Daily potential increase in HI	/day	0.019	0.036	0.017
Maximum HI		0.52	0.54	0.53
**THERMAL TIME REQUIREMENTS FROM:**				
Sowing to emergence	°Cd	100	50	70
Emergence to end of juvenile	°Cd	235	580	500
End of juvenile to floral initiation	°Cd	50	90	100
Floral initiation to flowering	°Cd	40	20	20
Flowering to start grain fill	°Cd	50	70	50
Start grain fill to end grain fill	°Cd	240	250	300
End grain fill to maturity	°Cd	60	20	100
Maturity to harvest ripe	°Cd	5	5	5

Model validation with the adjusted cultivar-specific parameters for the short-season grain legumes provided excellent agreement between simulated and observed values for crop phenology with RMSE values being equal or less than 2 days for the time of 50% flowering and 5 or less days for time to physiological maturity (Table 8). Time to maturity was simulated with less accuracy than flowering for all legumes, possibly reflecting the additive effects of errors simulating the intermediate flowering and grain fill stages.

**Table 8:** **Statistical criteria (root mean square error, RMSE) and observed range and mean for evaluating the phenological development (time to 50% flowering and physiological maturity) of short-season varieties of common bean, cowpea, and lablab**.

**Species**	**Time to…**	**Unit^a^**	**RMSE**		**Observed range**	**Observed mean**	***N***
			**Absolute value**	**% of mean observed**			
Bean	50% flowering	DAP	1.4	3.9	35–38	36.5	10
	Physiological maturity	DAP	3.6	4.8	71–79	75.0	10
Cowpea	50% flowering	DAP	1.0	1.7	57–58	59.5	10
	Physiological maturity	DAP	5.0	5.6	85–89	89.5	10
Lablab	50% flowering	DAP	1.6	2.5	62–64	63	10
	Physiological maturity	DAP	2.1	2.1	98–102	100	10

a *DAP–days after planting*.

For common bean the model performance was excellent, represented by very low RMSE values for biomass and grain yield of 12.4 and 11.9% of the observed mean respectively. The model efficiency was very good for common bean too. The accuracy of the model in predicting biomass and grain yield of cowpea and lablab was overall good, indicated by fairly low RMSE values ranging from 23 to 26% of the observed mean (Table 9) (Supplementary Material, Appendix 7).

**Table 9:** **Evaluation of the model performance in simulating grain and total biomass of common bean, cowpea, and lablab using statistical criteria (root mean square error, RMSE, and model efficiency, EF) as well as observed range and mean**.

**Species**		**Unit**	**RMSE**		**Observed range**	**Observed mean**	**EF**	***N***
			**Absolute value**	**% of mean observed**				
Bean	Total biomass	kg ha^−1^	370.2	12.4	1,762–3,741	2975.7	0.64	10
	Grain yield	kg ha^−1^	191.9	11.9	977–1,956	1610.7	0.65	10
Cowpea	Total biomass	kg ha^−1^	915.7	23.5	2,574–5,629	3902.9	0.18	10
	Grain yield	kg ha^−1^	508.7	26.0	1,384–3,061	1956.7	0.18	10
Lablab	Total biomass	kg ha^−1^	791.7	20.8	2,546–5,474	3810.8	0.08	10
	Grain yield	kg ha^−1^	436.9	25.1	1,234–2,352	1740.7	−0.47	10

Furthermore, the model was validated for changes in soil moisture in the soil profile, which remains particularly important to accurately simulate crop production in rainfed systems. The overall changes in soil moisture were represented well by the model simulations, but a comparatively high standard deviation of the observed data indicate a high degree of variability within the soil (for details see Supplementary Material, Appendix 6).

### Simulation of the agricultural potential and water use efficiency

#### Phenology

The phenological development of the different legumes varied along the Machakos–Makueni transect and was shortest for common bean (32–37 DAP) followed by cowpea and lablab (Appendix 8). With increased mean temperatures observed, the phenological development decreased along the transect with longest time to flowering and maturity in Katumani and shortest in Kampi ya Mawe.

#### Biomass and grain yield

**Figure 4:**
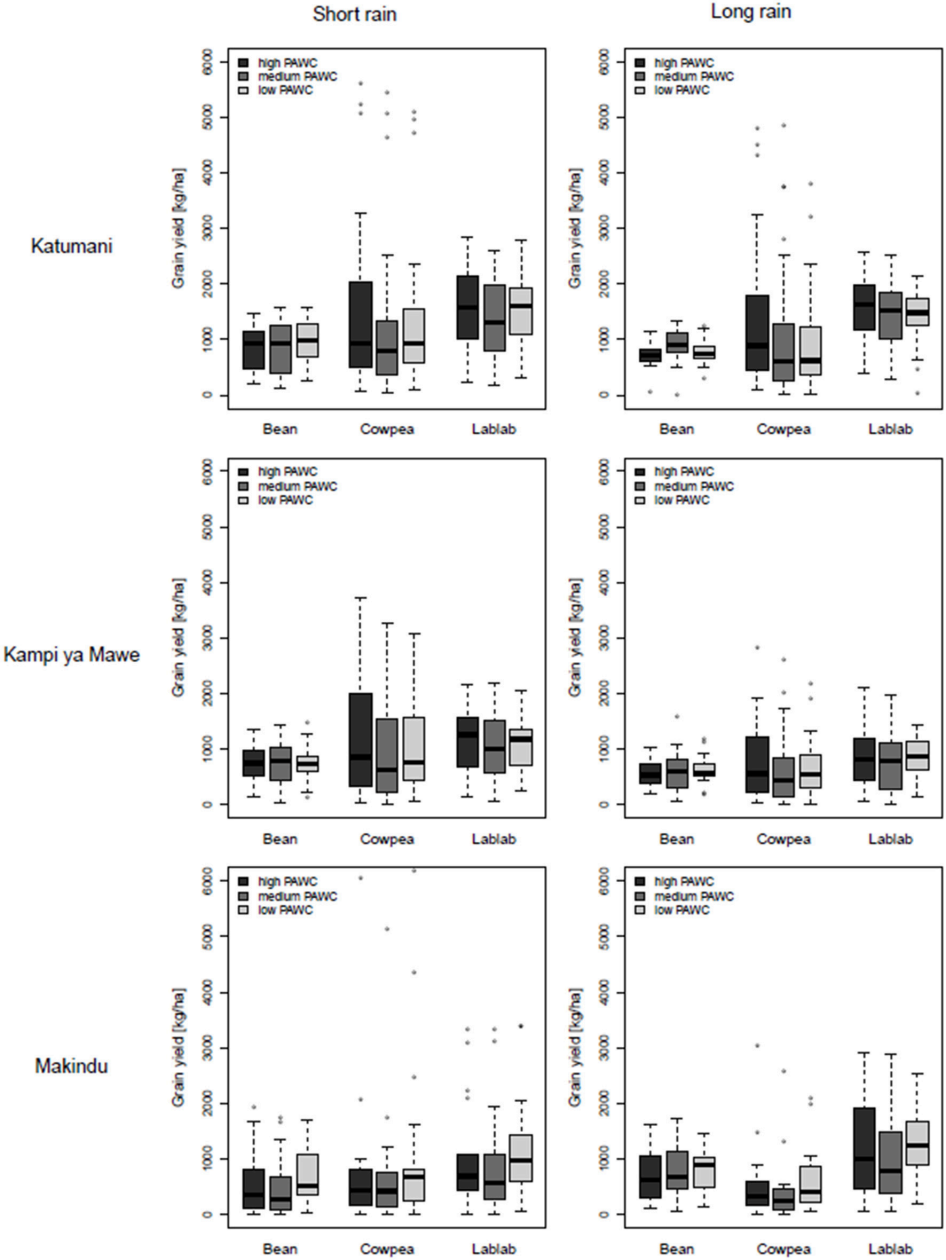
**Boxplots of simulated grain yields for common bean, cowpea, and lablab at different rainfall zones (Katumani, Kampi ya Mawe, and Makindu, Eastern Kenya) grown during the growing period of the short rain and the long rain on soils with different plant available water capacity (PAWC, high, medium, and low) based on results from the long-term simulation**.

In accordance with the results from the two trials, common bean displayed little response to in-crop rainfall, ranging from 50 to 400 mm, and the simulated median (50% quartile) of common bean grain yields was relatively stable at about 800 kg ha^−1^ at all sites and soils and for both growing periods of the short and the long rains (Figure 4, Appendix 9). The 25 and 75% quartile give a measure of the long-term variability and were only about plus minus 20–30% of the median common bean grain yield and, in particular, low for simulated yields during the growing period of the long rain in Katumani and Kampi ya Mawe (Figure 4). Only in 25% of the growing seasons in the last four decades, potential common bean yield exceeded 1,000 kg ha^−1^. However, with effective in-crop rainfall of less than 200 mm, comparatively high potential common bean grain yields were observed, even exceeding cowpea grain yield (Appendices 9, 10). Common bean grain yield can, therefore, be characterized as comparatively low but stable as there is a 50% chance to reach yields of about 1,000 kg ha^−1^ with about average in-season rainfall (200–400 mm). The site and the soil had relatively little impact on the simulated common bean grain yield.

In contrast, the observed yield variability was very high for cowpea, in particular at the high and medium rainfall zones Katumani and Kampi ya Mawe. Here, the inter-quartile range was as high as 1,500 kg ha^−1^ for the soil with a high PAWC and about 1,000 kg ha^−1^ for soils with medium to low PAWC, correlating with the high intra-seasonal rainfall variability at these sites (Figure 4). Cowpea grain yields of >3,000 kg ha^−1^ was possible in wet seasons with rainfall above 400 mm (Appendices 9, 10). At the low-rainfall zone in Makindu, the simulated cowpea grain yield as well as the probability to harvest more than 1,000 kg ha^−1^ was even lower than that of common bean, caused by relatively high water losses through crop transpiration.

**Figure 5:**
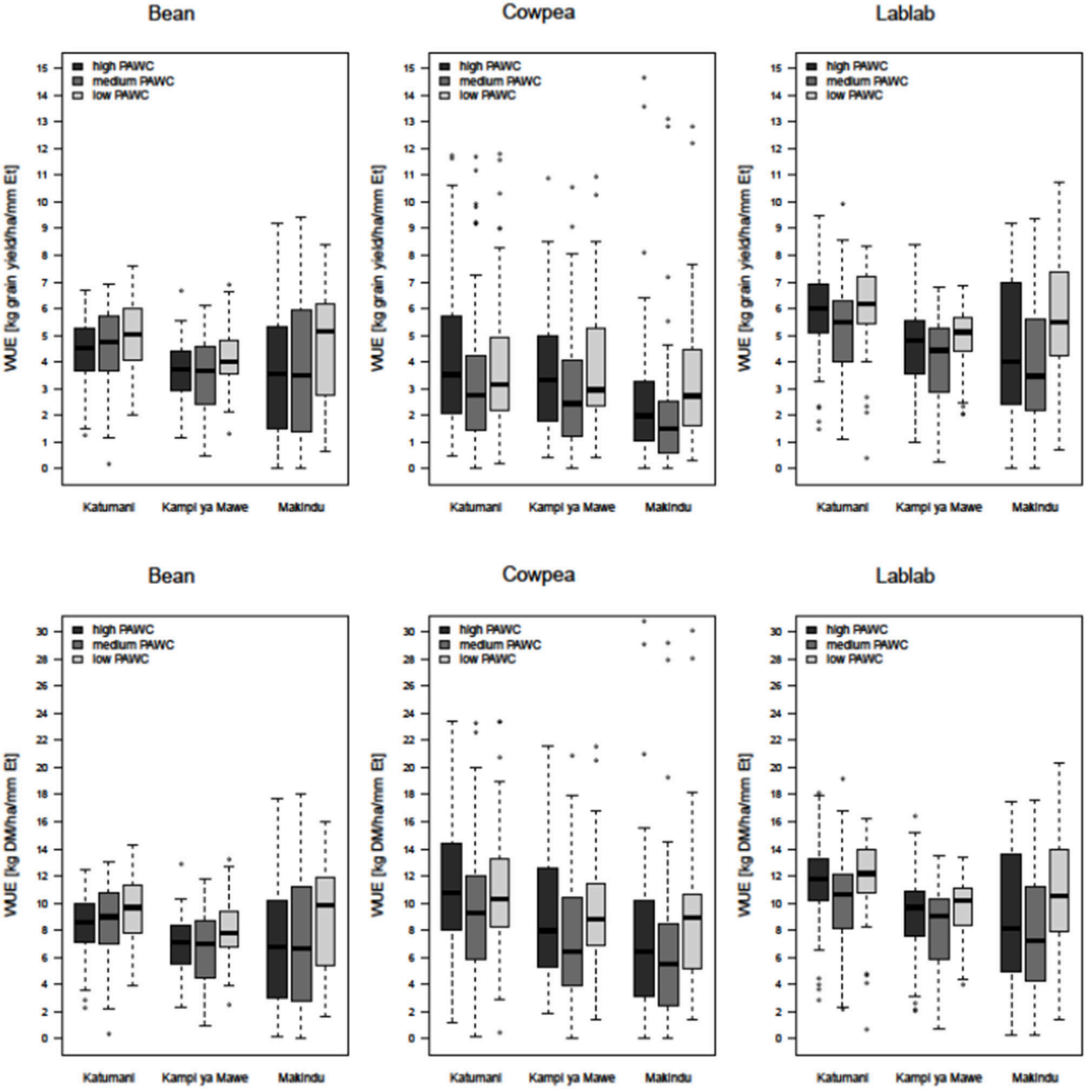
**Boxplots of simulated long-term average water-use efficiency (top:** kg grain yield ha^−1^ mm^−1^ E_t_; **bottom:** kg DM biomass ha^−1^ mm^−1^ E_t_)for common bean, cowpea, and lablab at different rainfall regions (Katumani, Kampi ya Mawe, and Makindu) for different soils (high plant available water capacity (PAWC), medium PAWC, low PAWC).

In comparison to cowpea, lablab was less responsive to effective in-crop rainfall, however, the simulated median yields were always highest in comparison to the other legumes (Figure 5). In particular, at the low-rainfall zone Makindu during the growing period of the long rain, there was still a 50% probability that lablab yields were above 1,500 kg ha^−1^ (Figure 4). However, it seemed that lablab cultivar used has a variety-specific threshold of 3,000 kg ha^−1^, which cannot be exceeded independent of the environmental conditions (Appendix 9). Consequently, the slope of yield increase with increased rainfall was less steep than observed for cowpea. At Katumani, simulated lablab grain yields at low levels of effective in-crop rainfall were generally higher in the growing period of the long rains in comparison to the short rains. Lablab might have benefited from a better usage of residual soil moisture from the short rain in comparison to the dry long rain season.

#### Soil evapotranspiration and crop transpiration

The simulations also showed that the amount of soil evaporation and crop transpiration for different soils and sites along the transect was not constant. In general, soil evaporation, crop transpiration and, consequently, evapotranspiration were lowest for common bean independent of soil and site, caused by the significantly shorter growing period and compact growth habit in comparison to cowpea and lablab. This is further reflected in the rather low LAI reached by common bean over the growing period (Supplementary Material, Appendix 5). But even if the growing period of cowpea was significantly shorter than that of lablab at most sites, crop transpiration was always highest for cowpea (data not shown), caused by the large crop canopy, wide surface coverage and relatively high biomass accumulated, further represented in very high maximum LAI values of up to five during the late vegetative growth period, flowering and early grain filling (Supplementary Material, Appendix 5). The small and bushy common bean transpired relatively little, between 36 and 51 mm on average, depending mainly on the rainfall zone. Lablab plants were larger than common bean but the compact and less spreading growth habit led to relatively low crop transpiration in comparison to cowpea, ranging from about 50 mm at Makindu to about 70 mm in Katumani. Soil evaporation was also correlated to the amount of seasonal rainfall. With increasing seasonal rainfall much more water was lost through soil evaporation. Relatively high biomass production and a good canopy soil coverage, however, reduced soil evaporation as observed for cowpea in comparison to lablab.

Even if the soil had no significant impact on the simulated legume grain yield at each individual site, a larger variation in cowpea and lablab grain yield was observed on clay soils (high PAWC) at the high and medium rainfall zones (Figure 4), indicating higher yields in the wetter seasons but also a greater risk of yield failure in drier seasons. At the low-rainfall zone Makindu, median cowpea and lablab yields were slightly higher on the sandy soil (low PAWC) instead, indicating a better availability of the scarce water on these soils in particular at low-potential areas. The effect of pre-season water storage on PAWC during the short rain season is negligible as the soils in semi-arid Eastern Kenya are usually completely dried out after the long dry period from July to October.

#### Water-use efficiency

The WUE_grain_ was not statistically different for common bean, cowpea and lablab at the medium and low-rainfall zones Kampi ya Mawe and Makindu (Figure 5). Nevertheless, median WUE_grain_ was always highest for lablab, but only statistically significantly higher at Katumani and always greater than 5 kg ha^−1^ mm^−1^ E_t_ for all soils. Whereas, the average WUE_grain_ of common bean and cowpea ranged from 3 to 4.5 kg ha^−1^ mm^−1^ E_t_ only and was even below 3 kg ha^−1^ mm^−1^ E_t_ at the low rainfall site Makindu for cowpea. The WUE in terms of biomass production was significantly higher for cowpea and lablab (8−12 kg ha^−1^ mm^−1^ E_t_) in comparison to common bean (6–8 kg ha^−1^ mm^−1^ E_t_). Moreover, average WUE_biomass_ was always higher at the high-rainfall site Katumani if compared to the low- and medium-rainfall sites. The site effect on WUE_biomass_ was very clear for cowpea. Furthermore, the inter-quartile range was increased from the high- to the low-rainfall site, particularly for common bean and lablab, indicating an increased variability with decreased seasonal rainfall. Surprisingly, average WUE_grain_ and WUE_biomass_ were always higher at the sandy soil with low PAWC in comparison to the clay soil with medium PAWC, in particular at the low-rainfall site Makindu, representing better water availability and allocation of the limited resource at these sites.

## Discussion

### Changes in growing season characteristics

The results of high season-to-season variation in the amount and distribution of rainfall, as well as decreased rainfall and increased temperatures in semi-arid Eastern Kenya, is in agreement with other studies from the same region (Rao and Okwach, 2005; Kabubo-Mariara and Karanja, 2007; Claessens et al., 2012). While naming the seasons may be confusing, the period known as the short rains generally receives more rain and is known to be more reliable than the period known as the long rains (Camberlin and Okoola, 2003; Rao and Okwach, 2005; Karanja, 2006). Consequently, the season of short rains is more important for agricultural activities in the area. Crop yields were, however, highly elastic in respect to changes in rainfall, including rainfall amount and distribution (Kabubo-Mariara and Karanja, 2007). It is expected that the temperature increase (Figure 1) might have a more severe impact on crop production as it accelerates crop development and ripening processes (Supplementary Material, Appendix 8). Cooper et al. (2009), for instance predict that an increase in temperature of 3°C will cause a mean decline of groundnut yield in Zimbabwe of 33% and pigeon pea yield in Kenya by 19%, mainly caused by faster and earlier maturity. The results from the simulation also indicate slightly reduced grain yields of all three short-season grain legumes at the site with the highest temperatures (Supplementary Material, Appendix 10). However, this might not be caused by higher mean temperatures and, consequently, accelerated ripening processes alone, as this may be a reaction to more extreme temperature events. Available crop varieties, including the tested short-season grain legume varieties, may therefore not be able to exhaust their physiological potential due to the shortened development time aligned with increased heat stress in the view of climate change (Figure 1). Fact is, changes in both rainfall pattern and temperatures can shift or even shorten traditional growing periods. The length and start of the growing period and, most importantly, its reliability, however, determine the suitability of a cropping strategy in a certain area, which is a fundamental indicator for site-specific yield potential (Cooper et al., 2009; Recha et al., 2013).

### Specific niches for different short-season grain legumes

The study revealed important differences in growth, development and resource use of legume species/varieties, emphasizing the suitability of specific characteristics and traits for different applications within the smallholder farming systems. In general, the studied short-season grain legumes seemed to follow the physiological strategy of drought escape (Vadez et al., 2012) as they flower and mature comparatively earlier than commonly grown maize crops (Supplementary Material, Appendix 8). Common bean flowered about five weeks after planting and was ready to harvest after ten weeks or less (Table 7). Consequently, water-potential yield of common bean was relatively stable (1,000 kg ha^−1^), independent of total in-crop rainfall and soil conditions (Figure 4). No responsiveness to increased water availability was observed and even at the low-potential site Makindu or at soils with low PAWC, grain yields were not significantly reduced. Many studies on legumes show that short-duration genotypes have higher and more stable yields than longer duration types (Turner et al., 2001; Vadez et al., 2012). However, the earliness decreases the overall yield potential of common bean. The fast development is compromising maximal biomass accumulation as a perquisite for grain production and the risk of reducing soil water to a level that will limit the reproductive growth. This is a rather conservative strategy, but might be advantageous in challenging environments with shortened cropping windows and high rainfall variability such as the low-potential site Makindu (Subbarao et al., 1995). This is also reflected in the observed and simulated WUE_grain_ of common bean, which is higher than cowpea, in particular for the low-potential site Makindu, confirming better water use and higher yields of common bean in challenging environments. These results further emphasize, that the availability of water during specific developmental stages are more important in determining final WUE_biomass_ and WUE_grain_ than the total water use alone (Zhang et al., 2000; Bell et al., 2012). At high potential sites, in contrast, common bean yields are not increased proportionally. Results show that the compact growth habit can also be disadvantageous, leading to a high share of water loss through soil evaporation, indicating an inadequate surface coverage and potential for improvement through plant density adjustments with increased water availability. As legumes can lose up to 60% of evapotranspiration in the form of soil evaporation (Zhang et al., 2000; Turner et al., 2001), the right selection of crop species and varieties in accordance with suitable management interventions (e.g., planting time, mulching) is important to improve water use and thereby the productivity of the cropping system. Consequently, in areas where water loss through soil evaporation is a major problem, any strategy involving fast canopy closure, early canopy interception or soil surface coverage techniques, will increase transpiration and thereby yield (Turner et al., 2001; Passioura and Angus, 2010).

This approach is characteristic for cowpea as the later flowering time allowed for an increased investment into pre-anthesis biomass accumulation. The vigorous and spreading growth habit (LAI >5) of cowpea further led to better surface cover, higher interception of radiation and, consequently, lower soil evaporation beneath the canopy in comparison to the more bushy common bean and lablab varieties. This strategy enabled cowpea to maximize its WUE in seasons with evenly distributed and above-average rainfall. In season, Where in-season dry spells are experienced or seasons are unexpected short, the high investment in increased pre-anthesis biomass is very risky and requires satisfying post-anthesis water supply in order to obtain high yields; otherwise transpiration requirements exceed water availability and cause stress and consequently reduced yields (Table 6 and Figures 4, 5). However, this rather risky strategy comes with the cost of increased crop transpiration and is rather disadvantageous in areas like semi-arid Eastern Kenya, where in-crop dry spells are common and the season length is highly variable. Therefore, the variance in both biomass and grain yield, as well as WUE_biomass_ and WUE_grain_, was generally large for legumes (Muchow, 1985; Zhang et al., 2000; Bell et al., 2012), and particularly high for cowpea at the high- and medium-potential sites Katumani and Kampi ya Mawe. However, in low-potential sites, such as Makindu where in-season dry spells are common and in-season rainfall is generally low, cowpea cannot exhaust its potential, and WUE and grain yield remained below those of common bean. WUE was in general lower at drier sites for all three legumes, presumably because a greater proportion of crop water use was lost as evaporation (Bell et al., 2012). Matching phenology with water availability and minimizing water loss through soil evaporation and crop transpiration, through the control of growth and development, are crop survival mechanisms in water-limited environments.

In general, lablab seemed to be best adapted to dry environments as the grain yield remained comparatively high at the low-potential site Makindu and in years with below-average rainfall, independent of the crop's comparatively long development time (Figure 4, Supplementary Material, Appendix 10). The canopy architecture is a good compromise between the right investment in biomass as a prerequisite to accumulate grain yield by minimizing water loss through soil evaporation and crop transpiration simultaneously. Another indicator for adaptation to semi-arid areas, including high rainfall variability and extreme temperatures, is the high WUE of lablab (Muchow, 1985). Even at the low-potential site Makindu, WUE_biomass_ and WUE_grain_ remained comparatively high (Figure 5). Lablab seemed to further benefit from its increased phenological plasticity enabling the crop to better adapt its phenological development to actual weather conditions—a strategy to escape water deficit through faster development, e.g., shortened grain-filling period. This highlights the suitability of lablab for cropping with increased uncertainties along the Machakos-Makueni transect in semi-arid Eastern Kenya. In contrast to the above discussed disadvantages of accelerated ripening aligned with increased temperatures, faster development caused by decreased water availability also highlights opportunities to avoid periods of stress and securing yield. Another advantageous feature contributing to this improved drought tolerance and comparatively high and stable yields in semi-arid environments might be the pubescent leaf surface of the lablab variety in comparison to the glabrous and dark green leaves of the cowpea variety. Clear-colored hairy leaves reflect more light, reduce the leaf surface temperature and, consequently, the crop transpiration (Subbarao et al., 1995). This is particularly important in view of increased temperatures to minimize the possible negative effects on crop growth and development. The lablab variety could continue to grow for a longer period of drought than cowpea, and achieve higher yields with less rainfall (Appendix 10).

### Modeling characteristics of short-season grain legumes

The HI as well as the daily increase in HI, together with the development times, are cultivar-specific parameters used to better calibrate APSIM for short-season grain legumes. Results show, that the parameters were selected well to account for the high-yielding and short-season characteristics of the selected legumes. The fit of observed and predicted phenological development, for instance, was excellent. The degree of agreement between observed and simulated biomass accumulation and yield production for common bean was very good, however, rather fair for cowpea and lablab. Nevertheless, the model accuracy for predicting biomass and yield production of cowpea and lablab is comparable to that achieved for other diverse legume species within the APSIM framework. Robertson et al. (2001) also reported fairly high RMSD values for predicting pigeonpea biomass and grain yield with 29.2 and 18.2% of the observed mean respectively. Similar was reported for the fababean grain yield with RMSD of 21% (Turpin et al., 2003). The RMSE values reported by Robertson et al. (2002) for mungbean, peanut, chickpea, and lucerene ranged from 22 up to 53% for biomass and grain yield.

The relatively satisfactory model performance for common bean in comparison to cowpea and lablab could be explained by the comparatively consistent and uniform growth and development—a results of centuries of targeted selection and breeding (Muñoz et al., 2017). The development time of common bean is significantly lower in comparison to cowpea and lablab. Consequently, there is less room to react to challenging environmental conditions. Furthermore, APSIM validation and calibration work has been pushed more intensively for common bean in comparison to other legumes, further contributing to better model performance of this species. Cowpea and lablab have a shorter breeding history and show a higher yield variability. Furthermore, there are still several characteristics of these grain legumes, which are not yet well-captured by the model, making accurate simulation difficult. The overlap of phenological stages characteristic for grain legumes, e.g., flowering and grain filling, is not well-represented and implicates problems in accurately predicting biomass accumulation and grain production. Furthermore, the phenlogical plasticity and observed ability of short-season grain legumes, in particular cowpea and lablab, to adapt their development time to water availability is not yet fully quantified and considered by crop growth models such as APISM. Particular difficult was the accurate prediction of density effects for lablab grain production; increased plant density led to vigorous biomass accumulation and comparatively low grain production. Another characteristic of short-season legumes is the ability to drop up to 50% of their leaves to compensate for an increased transpiration demand with increasing temperatures (Sennhenn, 2016) and/or decreased water availability without severe yield losses (Subbarao et al., 1995). Leaf nitrogen from senescent leaves is translocated toward the pods and used to accumulate grain nitrogen (Sanetra et al., 1998). This characteristic, however, is not yet captured in crop growth models, such as APSIM, and requires further calibration and validation. However, this feature provides legumes an advantage in comparison to other commonly grown cereal crops like maize and is further responsible for the comparatively high HI values (Siddique et al., 2001). Consequently, the improvement of legumes models still requires a better and deeper understanding of the physiological characteristics of these diverse legume species and comprehensive data to parameterize these functional relationships.

## Conclusion

In summary, comprehensive analysis of the long-term weather data confirmed that climate variability and the associated risks for crop production in semi-arid Eastern Kenya are particularly high and have an increasing trend. However, short-season grain legumes have great potential to address this challenge. The major traits of adaptation include early flowering and pod and seed set before the onset of terminal drought. In general, early phenology together with adapted canopy architecture allow for the optimization of better water use and greater partitioning of dry matter into seed. Results highlight that the studied short-season grain legumes have specific morphological, phenological, and physiological characteristics and follow different strategies to increase their production potential in challenging environments. Thus, showing a distinct suitability for specific cropping areas and purposes. The differences described here are characteristic for the selected varieties, which are the most commonly used in the study area. However, caution is needed to generalize these results as there are no true species differences. Nevertheless, the climate-smart site-specific utilization of these legumes, on the basis of the described results, offers promising options to design more resilient and productive farming systems in semi-arid Eastern Kenya.

## Author contributions

All four authors had substantial contribution to the conception, design, implementation, and analysis of the work. AS was mainly responsible to design and conduct field research as well as analyze and review the obtained data and draft the manuscript. AW accompanied the planning of the work from the early beginning as the former head of the department of Tropical Plant Production and Agricultural Systems Modelling (former Crop Production Systems in the Tropics), Georg-August-University Göttingen, whereas BM (former senior scientist at CIAT, Nairobi, Kenya) and DN (forage scientist at KALRO, Machakos, Kenya) mainly accompanied the work from the Kenyan site and facilitated the acquisition. All three, BM, DN, and AW, critically revised the analysis and data interpretation and approved the version, which was submitted for publication.

### Conflict of interest statement

The authors declare that the research was conducted in the absence of any commercial or financial relationships that could be construed as a potential conflict of interest.
