# Supplementary material for: Exploring Niches for Short-Season Grain Legumes in Semi-Arid Eastern Kenya — Coping with the Impacts of Climate Variability
Source: Front Plant Sci. 2017 May 9;8:699. doi: 10.3389/fpls.2017.00699 (PMC5422554; doi:10.3389/fpls.2017.00699)
Supplement: Supplementary file 1 [file DataSheet1.pdf]

Supplementary Material

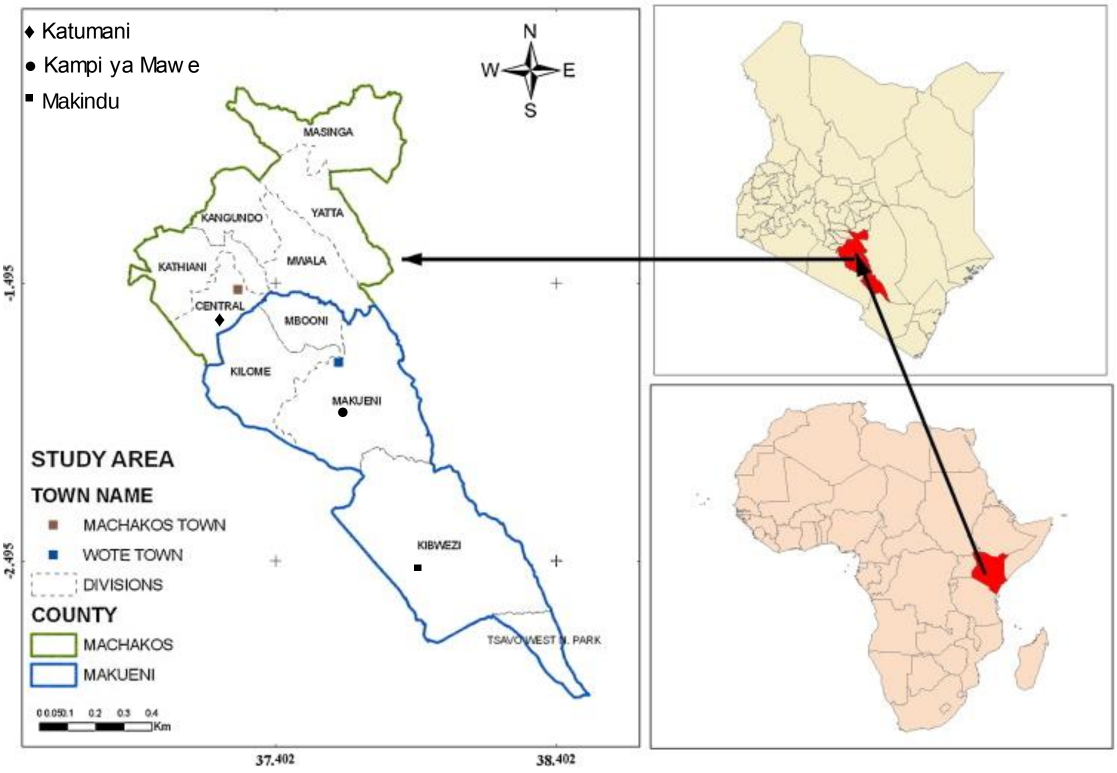

Appendix 1: Location map of the study are: Machakos and Makueni County, Eastern Kenya including the study sites: Katumani, Kampi ya Mawe and Makindu (adapted from Wambugu 2011).

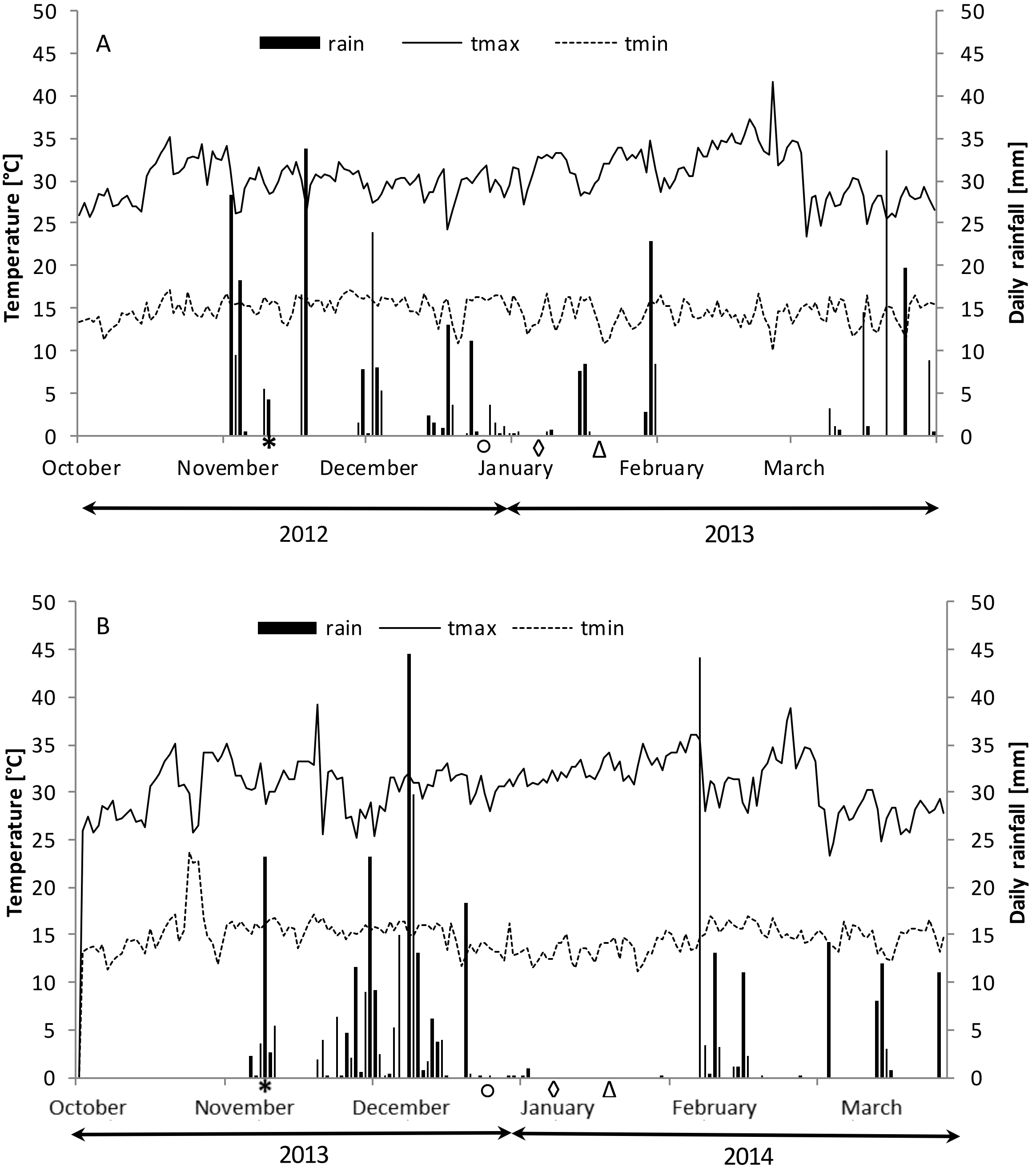

Appendix 2: Daily minimum and maximum temperatures, and rainfall during the growing periods of the short rains of (A) 2012/13 and (B) 2013/14 at KARI Katumani, Kenya.

* Planting date

○ Common bean harvest

◊ Cowpea harvest

Δ Lablab harvest

Appendix 3: Summary description of the treatments showing the plant density and water response trial at KARI Katumani, Machakos, Kenya during the short rains of 2012/13 and 2013/14.

Season	Species	Treatment plant density	Treatment water regime	Plant density [plants m^{-2}]	Irrigation [mm]	In-crop rainfall [mm]	Irrigation + rainfall [mm]
2012/13	Bean	low		5	270	156	426
		medium	fully irrigated	10	270	156	426
		high		20	270	156	426
			rainfed	10	0	156	156
		medium	partly irrigated	10	150	156	306
			fully irrigated	10	270	156	426
	Cowpea	low		5	300	190	490
		medium	fully irrigated	10	300	190	490
		high		8.33	345	190	535
			rainfed	10	0	190	190
		medium	partly irrigated	10	225	190	415
			fully irrigated	10	300	190	490
	Lablab	low		2.08	345	190	535
		medium	fully irrigated	4.17	345	190	535
		high		20	240	259	499
			rainfed	4.17	0	190	190
		medium	partly irrigated	4.17	210	190	400
			fully irrigated	4.17	345	190	535
2013/14	Bean	low		5	240	259	499
		medium	fully irrigated	10	240	259	499
		high		20	240	259	499
			rainfed	10	0	259	259
		medium	partly irrigated	10	60	259	319
			fully irrigated	10	240	259	499
	Cowpea	low		5	330	259	589
		medium	fully irrigated	10	330	259	589
		high		20	330	259	589
			rainfed	10	0	259	259
		medium	partly irrigated	10	180	259	439
			fully irrigated	10	330	259	589
	Lablab	low		3.3	345	339	684
		medium	fully irrigated	6.7	345	339	684
		high		13.3	345	339	684
			rainfed	6.7	0	339	339
		medium	partly irrigated	6.7	180	339	519
			fully irrigated	6.7	345	339	684

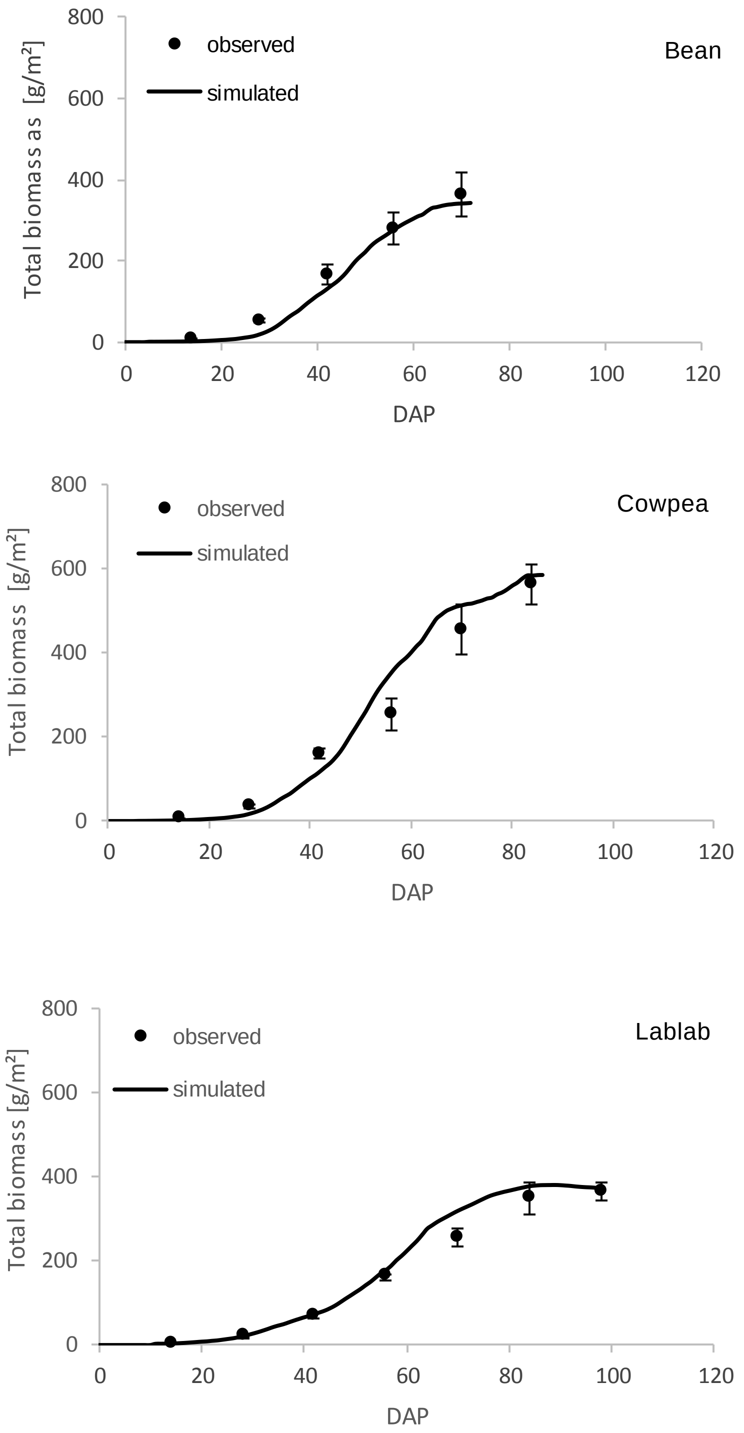

Appendix 4: Calibration of the APSIM model for common bean, cowpea and lablab showing observed and predicted values for total above ground biomass. Vertical bars represent standard deviation from observed data. DAP: days after planting.

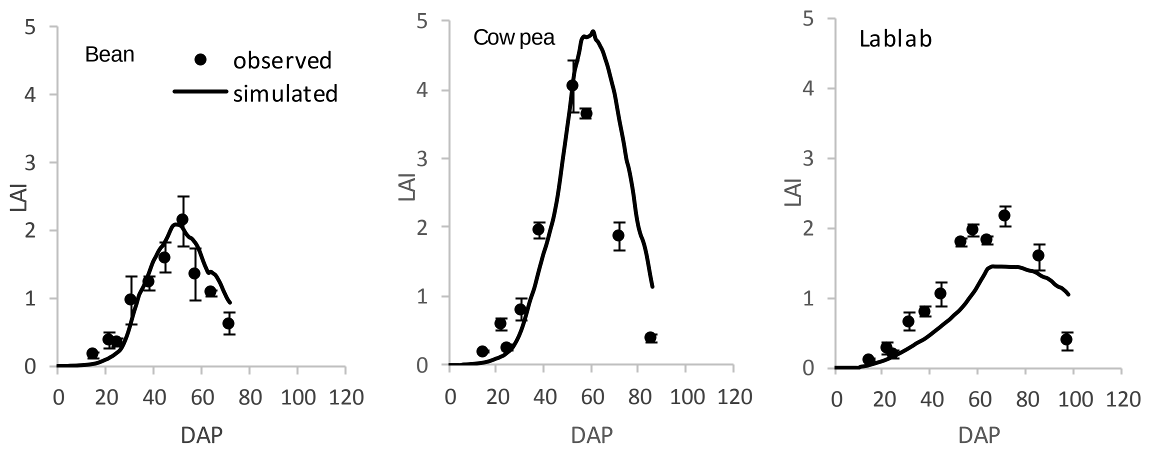

Appendix 5: Calibration of the APSIM model for common bean, cowpea and lablab showing observed and predicted values for leaf area index (LAI). Vertical bars represent standard deviation from observed data. DAP: days after planting.

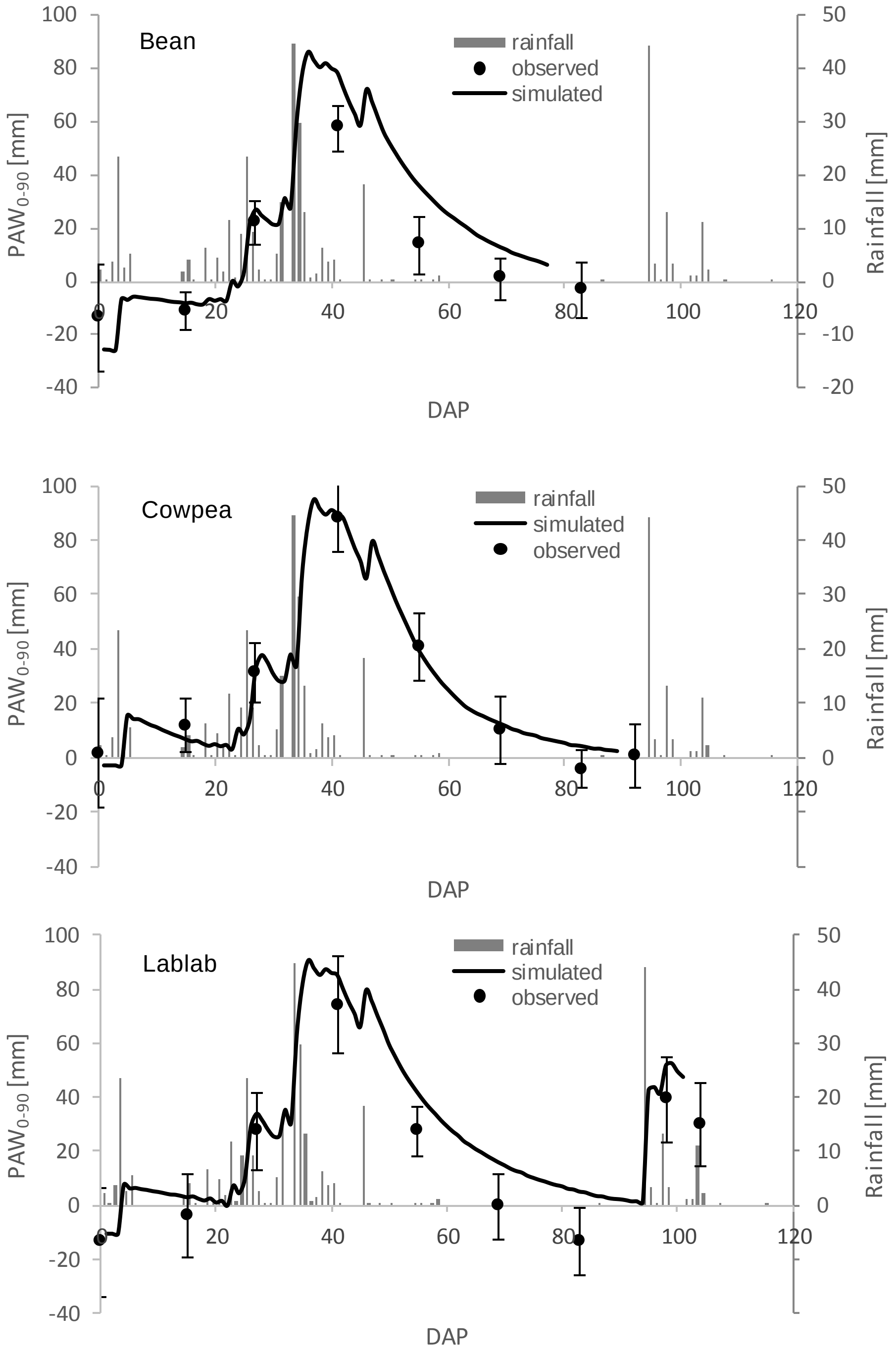

Appendix 6: Observed and simulated plant available water (PAW in mm) in the soil profile (0 – 90 cm) over the growing period for common bean, cowpea and lablab planted at medium density at rainfed conditions during the short rains of 2013/14. DAP: days after planting. PAW: plant available water.

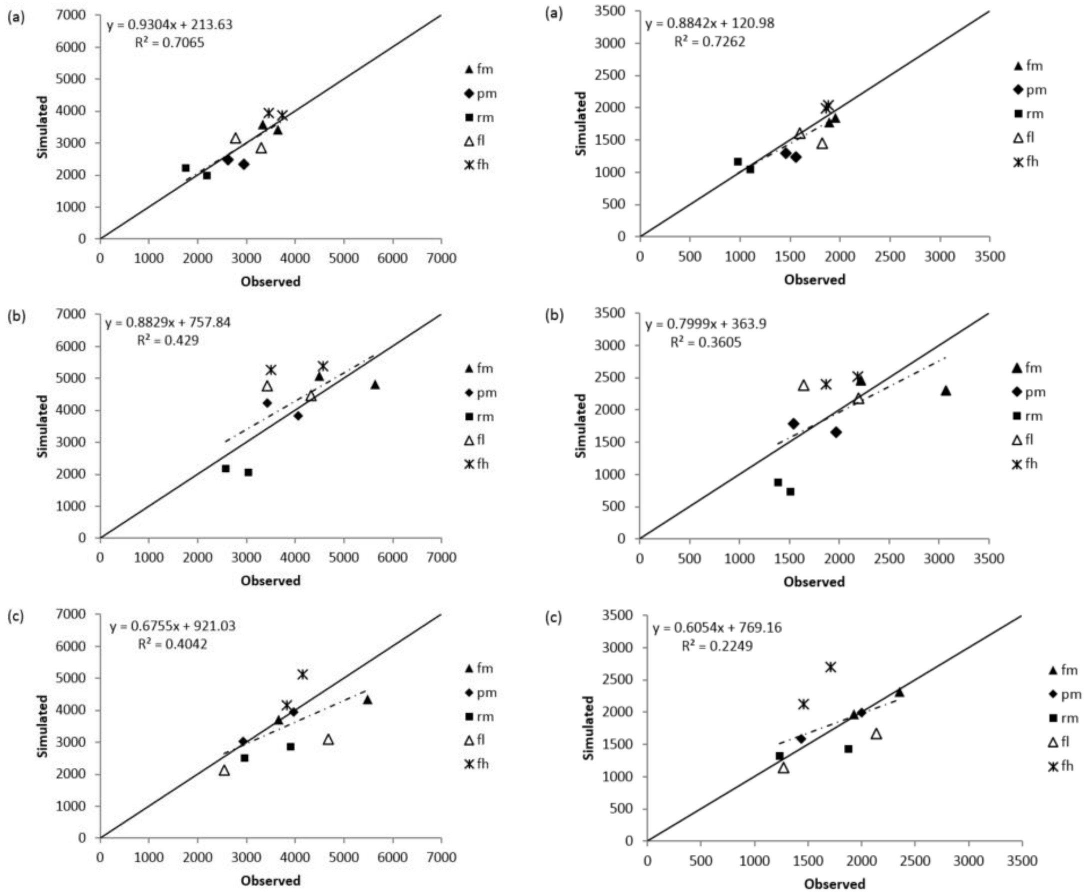

Appendix 7: Observed vs. simulated biomass in kg ha^{-1} (left) and grain yield in kg ha^{-1} for (a) common bean, (b) cowpea and (c) lablab for different plant density (experiment 1) and water regime (experiment 2) treatment combinations (fm: fully irrigated, medium density; pm: partly irrigated, medium density; rm: rainfed, medium density; fl: full irrigated, low density; fh: fully irrigated, high density) (Appendix 3).

Appendix 8: Simulated days to flowering and physiological maturity in days after planting (DAP) for common bean, cowpea and lablab grown in Katumani, Kampi ya Mawe and Makindu during the growing period of the short rain (SR) and long rain (LR).

	Katumani		Kampi ya Mawe		Makindu	
Species	SR	LR	SR	LR	SR	LR
			Flowering (DAP)			
Bean	37	36	32	35	34	33
Cowpea	80	84	60	57	67	69
Lablab	79	81	60	59	66	70
			Maturity (DAP)			
Bean	73	73	65	67	67	65
Cowpea	114	126	88	82	97	100
Lablab	123	143	97	94	95	112

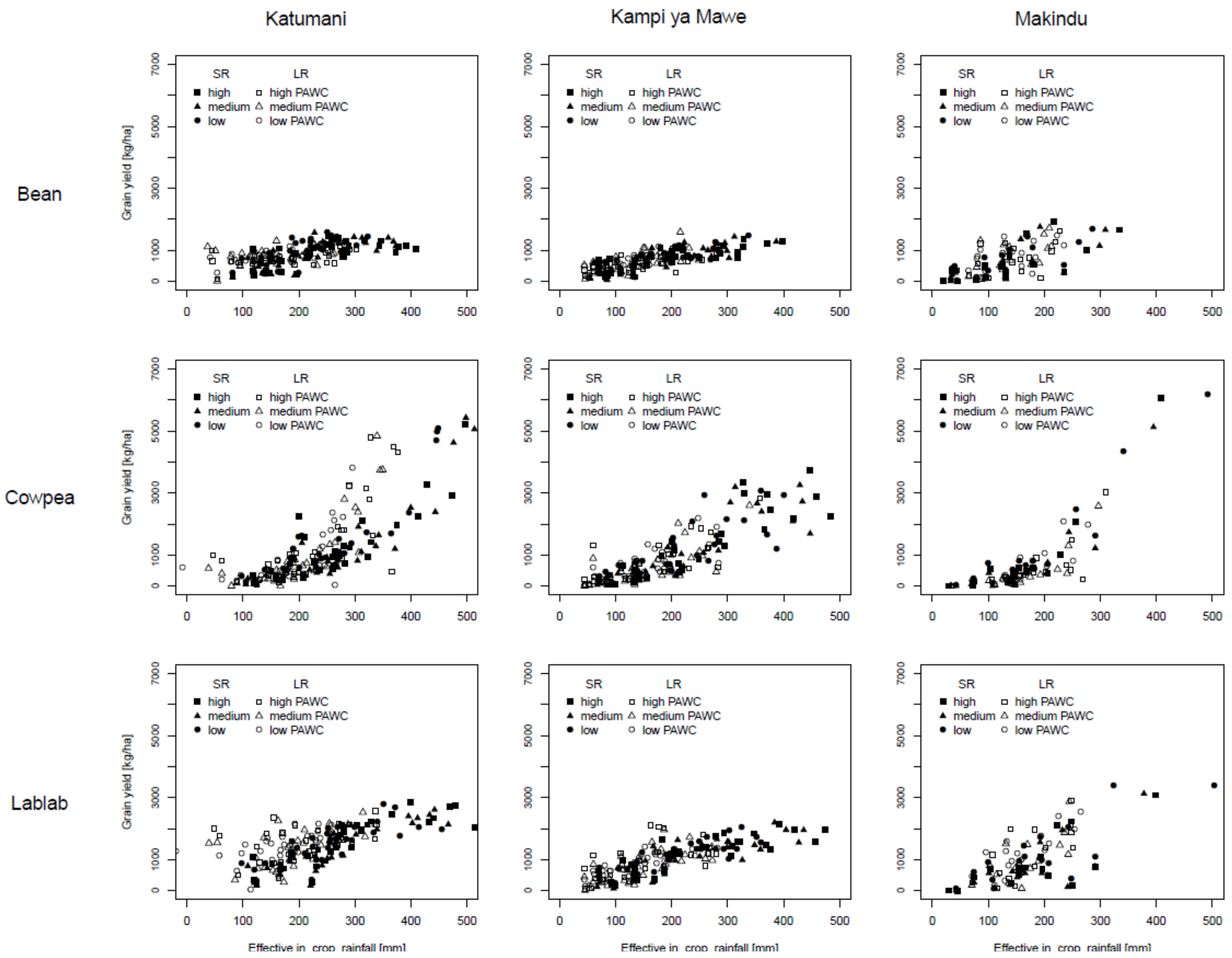

Appendix 9: Relationship between simulated legume grain yield kg ha^{-1} (top: common bean, middle: cowpea, bottom: lablab) and effective in-crop rainfall in mm at the high (Katumani, left), medium (Kampi ya Mawe, middle) and low rainfall zone (Makindu, right), simulated for soils with different plant available water capacity (PAWC; high, medium and low.

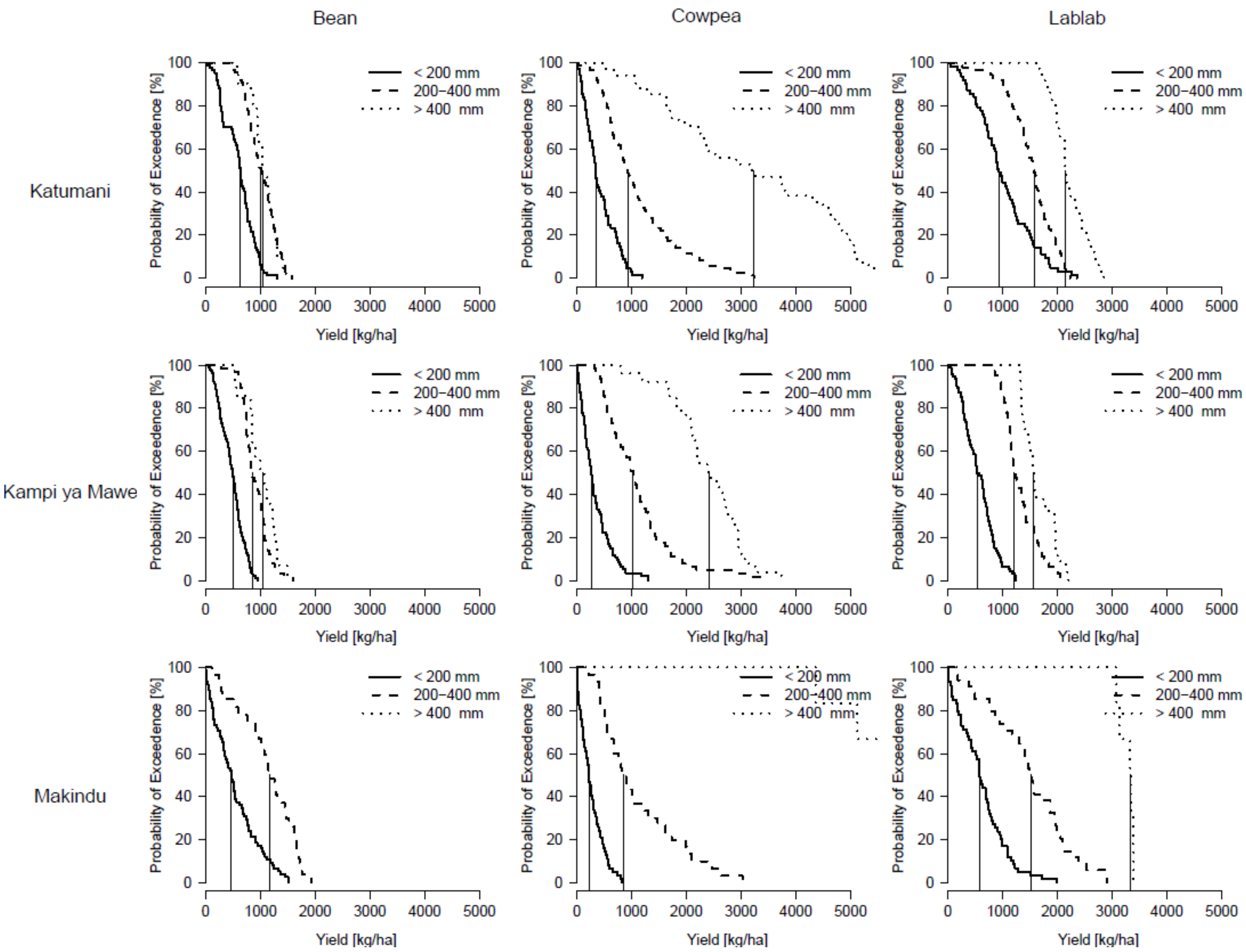

Appendix 10: Probability of exceedance of common bean, cowpea and lablab grain yield at different in-crop rainfall levels (<200, 200-400, >400 mm) at different rainfall zones (Katumani, Kampi ya Mawe and Makindu, Eastern Kenya) grown during the growing period of the short rain and the long rain based on results from the long-term simulation as described in the material and methods.
